# Hesperetin Ameliorates Inhibition of Neuronal and Oligodendroglial Cell Differentiation Phenotypes Induced by Knockdown of Rab2b, an Autism Spectrum Disorder-Associated Gene Product

**DOI:** 10.3390/neurolint15010025

**Published:** 2023-03-10

**Authors:** Yukino Kato, Remina Shirai, Katsuya Ohbuchi, Hiroaki Oizumi, Masahiro Yamamoto, Wakana Miyata, Tomoki Iguchi, Yoshihiro Mimaki, Yuki Miyamoto, Junji Yamauchi

**Affiliations:** 1 Department of Molecular Life Sciences, Tokyo University of Pharmacy and Life Sciences, Hachioji 192-0392, Tokyo, Japan; s179026@toyaku.ac.jp (Y.K.); s197073@toyaku.ac.jp (W.M.); miyamoto-y@ncchd.go.jp (Y.M.); 2 Tsumura Research Laboratories, Tsumura & Co., Inashiki 200-1192, Ibaraki, Japan; oobuchi_katsuya@mail.tsumura.co.jp (K.O.); ooizumi_hiroaki@mail.tsumura.co.jp (H.O.); hirokoma@h.email.ne.jp (M.Y.); 3 Department of Medicinal Pharmacognosy, Tokyo University of Pharmacy and Life Sciences, Hachioji 192-0392, Tokyo, Japan; iguchit@toyaku.ac.jp (T.I.); mimakiy@toyaku.ac.jp (Y.M.); 4 Department of Pharmacology, National Research Institute for Child Health and Development, Setagaya 157-8535, Tokyo, Japan; 5 Diabetic Neuropathy Project, Tokyo Metropolitan Institute of Medical Science, Setagaya 156-8506, Tokyo, Japan

**Keywords:** Rab2b, neuron, oligodendrocyte, neuronal marker, myelination marker, hesperetin

## Abstract

Autism spectrum disorder (ASD) is a central nervous system (CNS) neurodevelopmental disorder that includes autism, pervasive developmental disorder, and Asperger’s syndrome. ASD is characterized by repetitive behaviors and social communication deficits. ASD is thought to be a multifactorial disorder with a range of genetic and environmental factors/candidates. Among such factors is the rab2b gene, although it remains unclear how Rab2b itself is related to the CNS neuronal and glial developmental disorganization observed in ASD patients. Rab2 subfamily members regulate intracellular vesicle transport between the endoplasmic reticulum and the Golgi body. To the best of our knowledge, we are the first to report that Rab2b positively regulates neuronal and glial cell morphological differentiation. Knockdown of Rab2b inhibited morphological changes in N1E-115 cells, which are often used as the neuronal cell differentiation model. These changes were accomplished with decreased expression levels of marker proteins in neuronal cells. Similar results were obtained for FBD-102b cells, which are used as the model of oligodendroglial cell morphological differentiation. In contrast, knockdown of Rab2a, which is another Rab2 family member not known to be associated with ASD, affected only oligodendroglial and not neuronal morphological changes. In contrast, treatment with hesperetin, a citrus flavonoid with various cellular protective effects, in cells recovered the defective morphological changes induced by Rab2b knockdown. These results suggest that knockdown of Rab2b inhibits differentiation in neuronal and glial cells and may be associated with pathological cellular phenotypes in ASD and that hesperetin can recover their phenotypes at the in vitro level at least.

## 1. Introduction

Rab proteins belong to a large family of small GTPases and play essential roles as regulators of cellular membrane organization and intracellular trafficking in mammals and other eukaryotes [1,2,3,4]. Rab2 subfamily members are composed of Rab2a and Rab2b molecules. Rab2 proteins are involved in the regulation of retrograde transport between the endoplasmic reticulum (ER) and the Golgi body in many types of cells, including neuronal cell types. They are also involved in an autophagic pathway, as well as recycling around the Golgi body. These roles have been established in model animals such as *Drosophila melanogaster* and *Caenorhabditis elegans* [1,2,3,4]. Similar to other GTPases, Rab2a and Rab2b become active GTP-bound states whose forms are generated by guanine nucleotide exchange factors (GEFs) [1,2,3,4]. They also become inactive GDP-bound forms. The reactions are mediated by GTPase-activating proteins (GAPs). In the inactive states, they often reside in the cytoplasm, whereas in the active states, they translocate to the membrane surfaces to direct other coat proteins of the membrane ones [1,2,3,4].

Autism spectrum disorder (ASD) is a neurodevelopmental disorder that includes autism, pervasive developmental disorder, Asperger’s syndrome, and early-onset schizophrenia [5,6,7,8,9,10]. ASD is characterized by repetitive behaviors and impaired social communication. It is thought that ASD is multifactorial in nature, involving a range of genetic, epigenetic, and environmental factors. ASD has very diverse intrinsic factors [5,6,7,8]. In addition to neuronal tissue disorganization observed in the post-mortem tissue of ASD patients, gray and white matter disorganization is also exhibited, which implies that glial cells, especially myelin sheath–forming oligodendrocytes (also called oligodendroglial cells), are defectively organized in the brain structures [5,6,7,8]. In addition, ASD patients exhibit an increased number of neurons and other glial cells, such as astrocytes and microglial cells, reminiscent of immature or defective differentiating cell phenotypes with undifferentiating and/or proliferating neuronal and glial cells [5,6,7,8].

Among the intrinsic factors of ASD is the rab2b gene (Refs. [5,6,7,8]; also see the Harmonizome website, https://maayanlab.cloud/Harmonizome/, accessed on 10 January 2023). The Rab2b gene is localized in chromosome 8q12.1 to 8q12.2 [5,6,7,8]. Chromosome 8q12.1 to 8q12.2 involves ASD-associated chromodomain helicase DNA binding protein 7 (CHD7). It is suggested that genome alternations in 8q12.1 to 8q12.2 are critically linked to ASD [5,6,7,8]. However, the long-term question of whether loss-of-function of the rab2b gene is actually linked to defective neuronal and/or glial cell differentiation like the neuronal and oligodendroglial disorganization observed in ASD patients remains to be clarified. Here, for the first time, we describe the effects of knockdown of Rab2b on morphological changes in N1E-115 cells, which are often utilized as the model of neuronal differentiation [11,12], and in FBD-102b cells, which are used as the model of oligodendroglial differentiation [11,12]. We also describe the effects of knockdown of Rab2a or Rab4b, the Rab2b homologous gene products [1,2,3,4]. Furthermore, we find that hesperetin, a citrus flavonoid with multiple cellular protective effects [13,14,15,16], recovers defective cellular phenotypes induced by Rab2b knockdown. The results suggest how ASD-associated Rab2b can affect neuronal and oligodendroglial differentiation, suggesting a possible application of a flavonoid hesperetin for ASD at the molecular and cellular biological levels.

## 2. Materials and Methods

### 2.1. Antibodies, siRNAs, and DNA Primers

Key materials, such as antibodies, are listed in Table 1, Table 2, Table 3, Table 4, Table 5 and Table 6.

### 2.2. DNA Amplification

Total RNAs were extracted using Isogen, in accordance with the manufacturer’s instructions (Nippon Gene, Tokyo, Japan). Reverse transcription-polymerase chain reaction (RT-PCR) was performed in accordance with the manufacturer’s instructions (Takara Bio, Kyoto, Japan). The PrimeScript RT Master Mix kit was utilized to synthesize cDNAs from total RNA. Gflex DNA polymerase was utilized for DNA amplification. PCR (28–36 cycles) was performed at 98 °C for 0.2 min (denaturation reaction), 56 to 65 °C for 0.25 min (annealing reaction), and 68 °C for 0.5 min (extension reaction). Agarose gel electrophoresis was performed in accordance with the manufacturer’s instructions (Nacalai Tesque, Kyoto, Japan). The PCR products were applied to 1 to 2% agarose gels.

### 2.3. Cell Culture, Differentiation, and siRNA Transfection

Mouse neuronal N1E-115 cells (provided by Dr. Shiokawa, Tokyo University of Science, Chiba, Japan) were cultured on normal cell culture dishes (Greiner, Oberösterreich, Germany) in Dulbecco’s modified Eagle medium (DMEM, Nacalai Tesque) containing 10% heat-inactivated fetal bovine serum (FBS, Thermo Fisher Scientific, Waltham, MA, USA) and PenStrep (Thermo Fisher Scientific) in 5% CO_2_ at 37 °C [11,12]. To induce differentiation, N1E-115 cells were cultured in DMEM containing PenStrep in 5% CO_2_ at 37 °C for 3 days. Cells with more than one-cell-body-length processes were classified into category 1; cells with more than two-cell-body-length processes were classified into category 2; and cells with more than three-cell-body-length processes were classified into category 3. Cells belonging to category 3 were considered to be fully differentiated cells. Cells were transfected with the respective plasmids using a ScreenFect siRNA transfection kit (Fujifilm, Tokyo, Japan), according to the manufacturer’s instructions. The medium was generally replaced 4 h after transfection and was generally used for 48 to 72 h for biochemical experiments.

FBD-102b cells (provided by Dr. Tomo-Oka, Tokyo University of Science), which belong to a mouse oligodendroglial precursor cell line [11,12], were cultured on culture dishes in DMEM/F-12 mixed medium (Nacalai Tesque) containing 10% FBS and PenStrep in 5% CO_2_ at 37 °C. To induce differentiation, cells were cultured on polylysine (Nacalai Tesque)-coated cell culture dishes in a culture medium without FBS until 3 days in 5% CO_2_ at 37 °C. Cells with primary processes were classified as category 1; cells with secondary processes branched from the primary process were classified as category 2; and cells with third processes branched from the second process or with widespread plasma membranes were classified as category 3. Category 2 corresponded to these intermediate phenotypes, and category 3 was considered to be fully differentiated cells. Cell morphologies in arbitrary square fields were captured using microscopic systems equipped with iNTER LENS (Micronet, Inc., Saitama, Japan) and incorporated using Adobe Photoshop software (Adobe, San Jose, CA, USA).

Cells were transfected with the respective synthesized 21-mer siRNAs with dTdT (see Table 1 for siRNA sequences) using a ScreenFect siRNA transfection kit (Fujifilm) in accordance with the manufacturer’s instructions. The medium was replaced 4 h after transfection, and cells were further cultured for experiments in the presence or absence of hesperetin (Santa Cruz Biotechnology, Santa Cruz, CA, USA). Less than 5% of attached cells contained incorporated trypan blue (Nacalai Tesque) at 48 h after transfection.

### 2.4. Denatured Polyacrylamide Gel Electrophoresis and Immunoblotting

Cells were lysed in lysis buffer (50 mM HEPES-NaOH, pH 7.5, 150 mM NaCl, 20 mM MgCl_2_, 1 mM dithiothreitol, 1 mM phenylmethane sulfonyl fluoride, 1 μg/mL leupeptin, 1 mM EDTA, 1 mM Na_3_VO_4_, 10 mM NaF, and 0.5% NP-40) [11,12]. For denaturing conditions, their supernatants were denatured in a denaturing sample buffer (Nacalai Tesque). The samples were separated into 8 to 12% sodium dodecyl sulfate–polyacrylamide gels (Nacalai Tesque). The electrophoretically separated proteins were transferred to PVDF membranes (Fujifilm), blocked with Blocking One (Nacalai Tesque), and immunoblotted using primary antibodies, followed by peroxidase-conjugated secondary antibodies (MBL, Tokyo, Japan). A CanoScan LiDE400 image scanner (Canon, Tokyo, Japan) captured the peroxidase-reactive bands on X-ray films (Fujifilm). Quantified immunoreactive bands were compared with the control’s immunoreactive bands, which were set to 100%, using Image J software (NIH, Bethesda, MD, USA).

### 2.5. Statistics

From separate experiments, values are expressed using standard deviation (SD). For intergroup comparisons, the unpaired Student’s *t*-test was utilized (Excel software, Microsoft, Redmond, WA, USA). The one-way analysis of variance analyses was followed by Fisher’s protected least significant difference test (StatPlus software, AnalystSoft, Walnut, CA, USA). Statistically significant values were considered as *p* < 0.05.

### 2.6. Ethics

The Tokyo University of Pharmacy and Life Sciences Gene Committee and the Tokyo University of Pharmacy and Life Sciences Animal Care Committee approve cell and gene recombination techniques. Their approved numbers are LS28-20 and LSR3-011.

## 3. Results

### 3.1. Knockdown of Rab2b but Not Rab2a Specifically Inhibits Neuronal Morphological Differentiation

**Figure 1:**
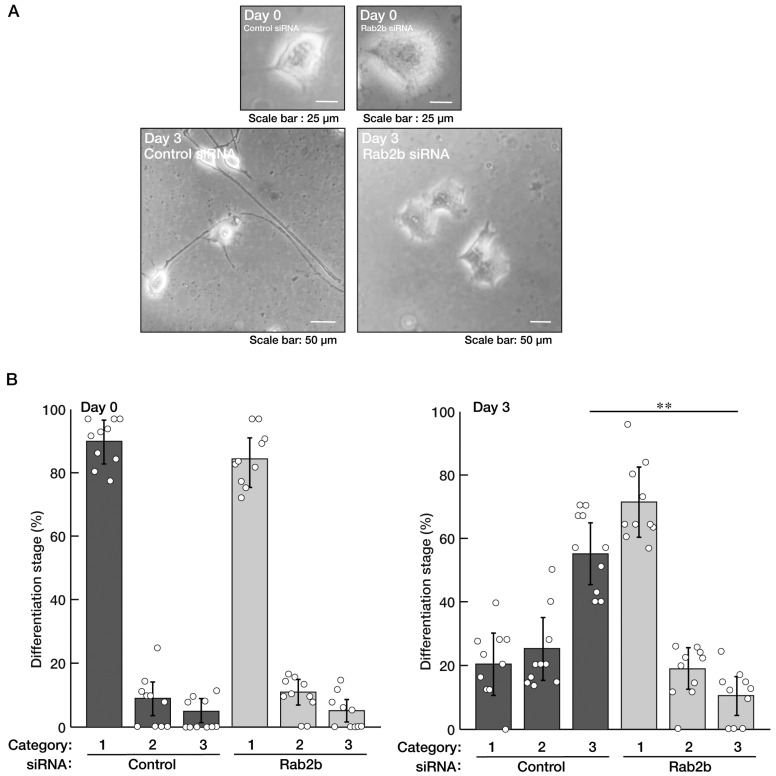
Knockdown of Rab2b decreases neuronal morphological differentiated phenotypes. (**A**,**B**) N1E-115 cells were transfected with a control (luciferase) or Rab2b siRNAs. Transfected cells were allowed to be cultured for 0 or 3 days following the induction of differentiation. Their cell morphologies were divided into 3 categories and statistically depicted as graphs (** *p* < 0.01; n = 10 fields). A differentiated phenotype as elongation of neuronal processes is shown to be promoted in the order of categories 1 to 3. White circles show percentages in the respective independent experiments.

**Figure 2:**
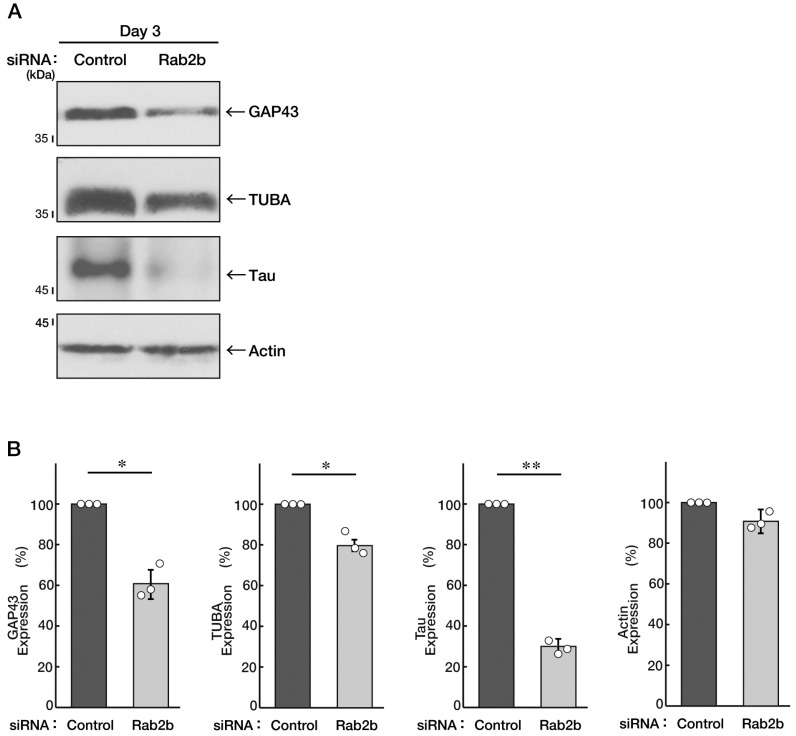
Knockdown of Rab2b decreases expression levels of neuronal differentiation marker proteins. (**A**,**B**) N1E-115 cells were transfected with a control or Rab2b siRNAs. Transfected cells were allowed to be cultured for 3 days following the induction of differentiation. Each cell lysate was collected for an immunoblotting for an antibody against GAP43, TUBA, Tau, or actin. Immunoreactive bands were quantitatively calculated compared to the respective controls (100%) and statistically depicted as graphs (** *p* < 0.01, * *p* < 0.05; n = 3 blots). White circles show percentages in the respective independent experiments.

Neuronal tissue disorganization is often observed in the brains of ASD patients [5,6,7,8]. Therefore, to verify the possibility that Rab2b, the gene product of an ASD intrinsic factor [5,6,7,8], is involved in the regulation of neuronal cell differentiation, we knocked down Rab2b using the specific siRNA (Figure S1) in N1E-115 cells and tried to examine the role of Rab2b in neuronal morphological differentiation. The knockdown resulted in inhibiting the extension of neural processes following the induction of differentiation (Figure 1A,B). The phenotypes were accompanied by decreased expression levels of neuronal differentiation marker proteins, growth-associated protein 43 (GAP43), brain-rich alpha 1 type tubulin (TUBA), and Tau as neuronal cell-rich microtubule-associated protein (Figure 2A,B), indicating the role of Rab2b in neuronal morphological differentiation. In contrast, control actin proteins were comparable in control- and Rab2b-knocked down cells.

**Figure 3:**
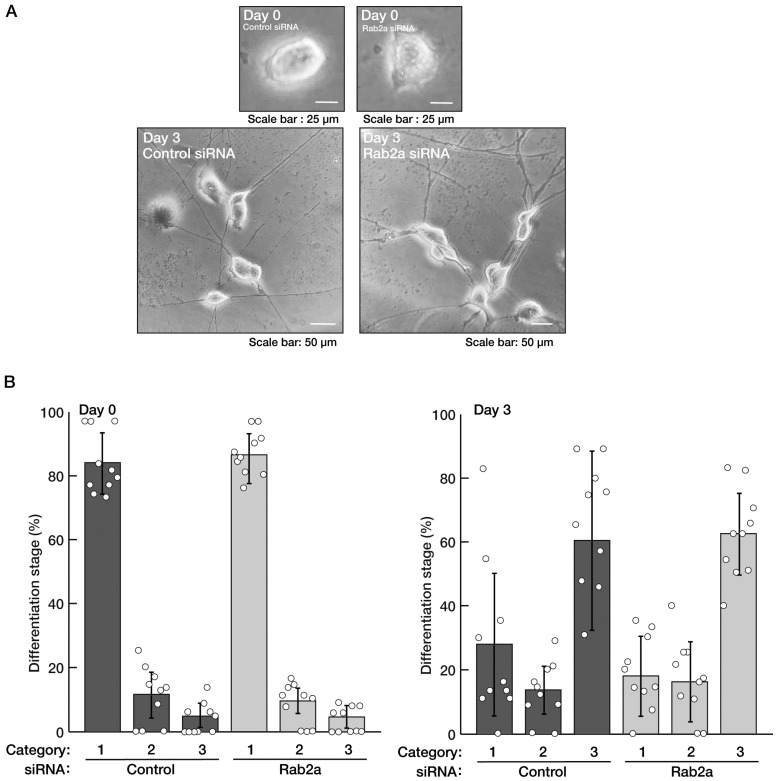
Knockdown of Rab2a does not significantly change morphologically differentiated phenotypes. (**A**,**B**) N1E-115 cells were transfected with a control or Rab2a siRNAs. Transfected cells were allowed to be cultured for 0 or 3 days following the induction of differentiation. Their cell morphologies were divided into 3 categories and statistically depicted as graphs (n = 10 fields). White circles show percentages in the respective independent experiments.

**Figure 4:**
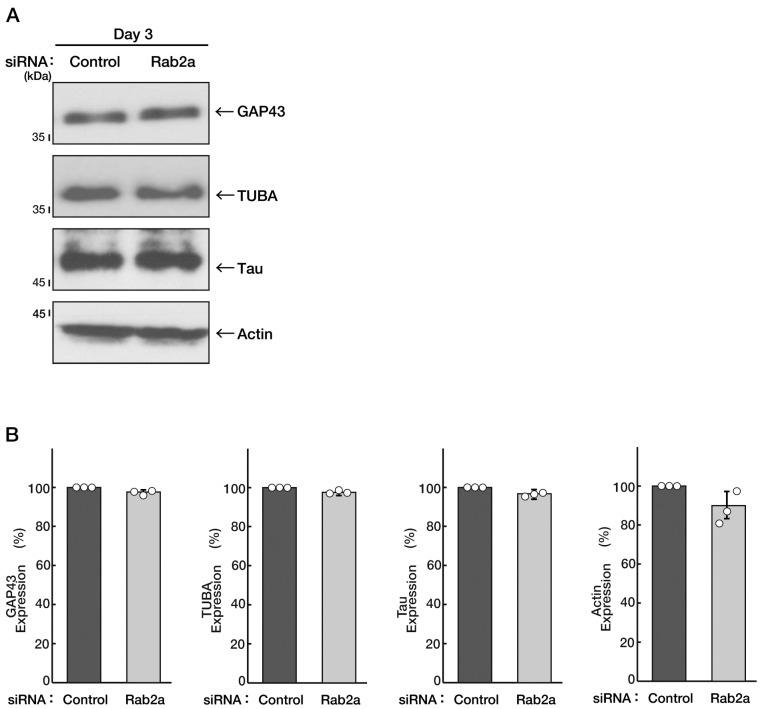
Knockdown of Rab2a does not significantly change expression levels of marker proteins. (**A**,**B**) N1E-115 cells were transfected with a control or Rab2b siRNAs. Transfected cells were allowed to be cultured for 3 days following the induction of differentiation. Each cell lysate was collected for an immunoblotting for an antibody against GAP43, TUBA, Tau, or actin. Immunoreactive bands were quantitatively calculated compared to the respective controls (100%) and statistically depicted as graphs (n = 3 blots). White circles show percentages in the respective independent experiments.

Next, we checked whether Rab2a, the Rab2b homologous gene product [1,2,3,4], also contributes to neuronal morphological differentiation. We transfected siRNA specific for the control or Rab2a into cells. Knockdown of Rab2a did not have a significant effect on neural process extension, being compared with control knockdown (Figure 3A,B). The results also reflected the expression levels of marker proteins (Figure 4A,B). Similar results were obtained in the case of the knockdown of Rab4b (Figure S2), the Rab2b/a homologous gene product (Figures S3 and S4) [1,2,3,4]. There were no obvious transcripts of Rab4a [1,2,3,4] in cells. These results suggest that Rab2b of the Rab2 subfamily proteins specifically mediates neuronal morphological differentiation.

### 3.2. Hesperetin Recovers an Inhibitory Effect of Rab2b Knockdown on Neuronal Morphological Differentiation

**Figure 5:**
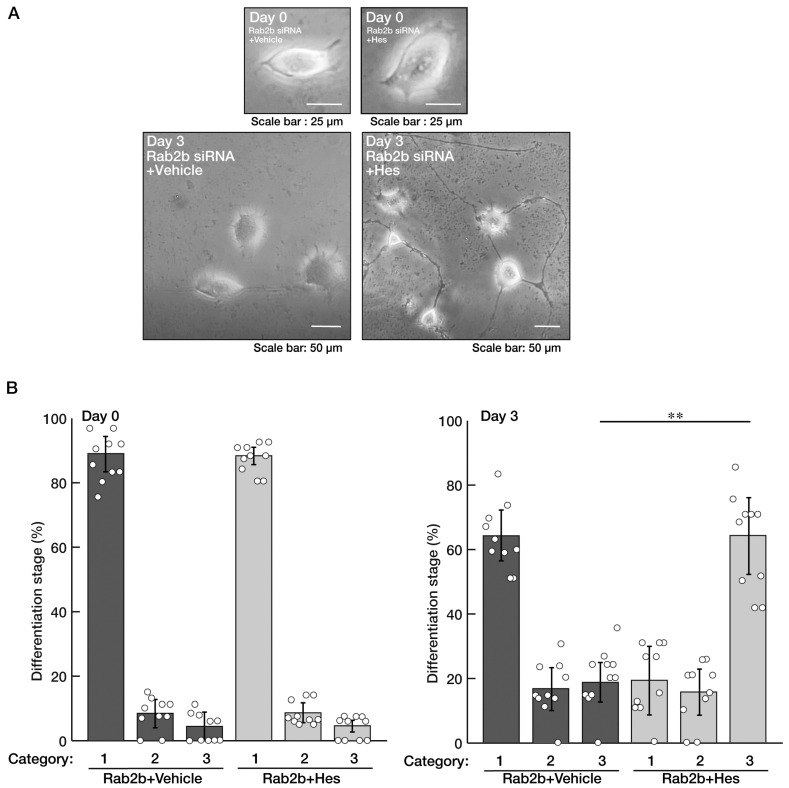
Hesperetin recovers decreased differentiated phenotypes induced by Rab2b knockdown. (**A**,**B**) N1E-115 cells were transfected with Rab2b siRNAs and treated with a control vehicle or hesperetin (Hes, 1 μM) for 0 or 3 days. Their cell morphologies were divided into 3 categories and statistically depicted as graphs (** *p* < 0.01; n = 10 fields). White circles show percentages in the respective independent experiments.

**Figure 6:**
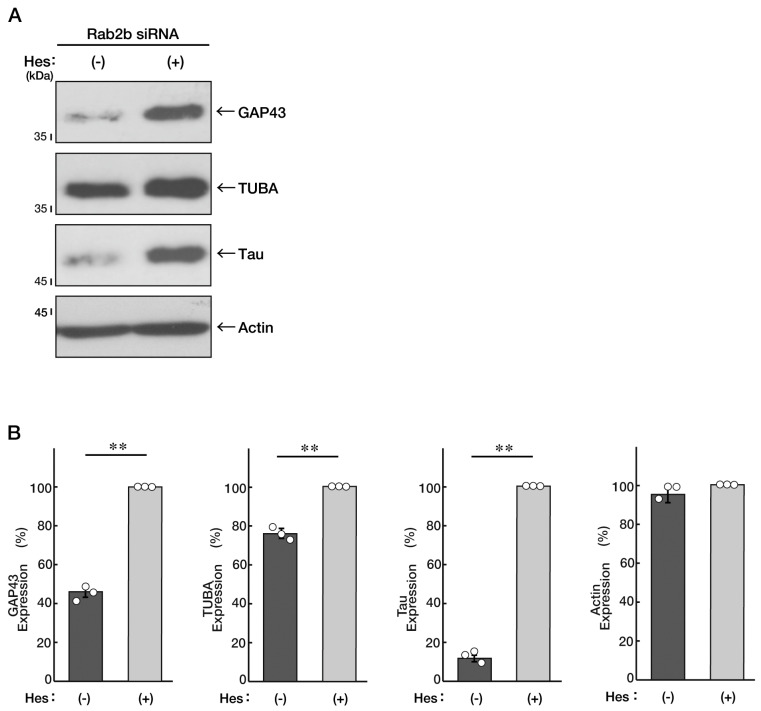
Hesperetin recovers decreased marker protein levels induced by Rab2b knockdown. (**A**,**B**) N1E-115 cells were transfected with Rab2b siRNAs and treated with a control vehicle (minus hesperetin [minus Hes]) or hesperetin (plus Hes, 1 μM) for 3 days. Each cell lysate was collected for an immunoblotting for an antibody against GAP43, TUBA, Tau, or actin. Immunoreactive bands were quantitatively calculated compared to the respective controls (100%) and statistically depicted as graphs (** *p* < 0.01; n = 3 blots). White circles show percentages in the respective independent experiments.

We asked whether hesperetin [13,14,15,16], a flavonoid with multiple neuronal protective effects, recovers the inhibition of differentiation induced by knockdown of Rab2b in N1E-115 cells. We treated the control vesicle, or hesperetin, with Rab2b-knocked down cells. Treatment with hesperetin resulted in recovering the effect of Rab2b knockdown on differentiation and restored the cellular phenotypes extending neuronal processes (Figure 5A,B). Additionally, hesperetin recovered the decreased expression levels of marker proteins induced by Rab2b knockdown (Figure 6A,B).

### 3.3. Knockdown of Rab2b Inhibits Oligodendroglial Morphological Differentiation, as Recovered by Hesperetin

**Figure 7:**
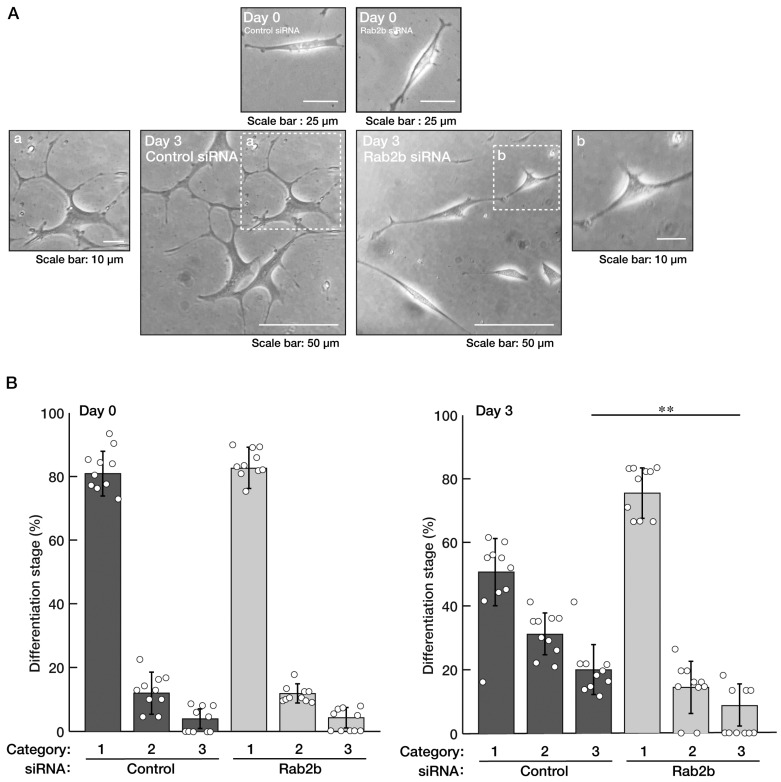
Knockdown of Rab2b decreases morphologically differentiated phenotypes in FBD-102b cells. (**A**,**B**) FBD-102b cells were transfected with a control or Rab2b siRNAs. Transfected cells were allowed to be cultured for 0 or 3 days following the induction of differentiation. Their cell morphologies were divided into 3 categories and statistically depicted as graphs (** *p* < 0.01; n = 10 fields). A differentiated phenotype as complexity of branches is shown to be increased in the order of categories 1 to 3. White circles show percentages in the respective independent experiments.

**Figure 8:**
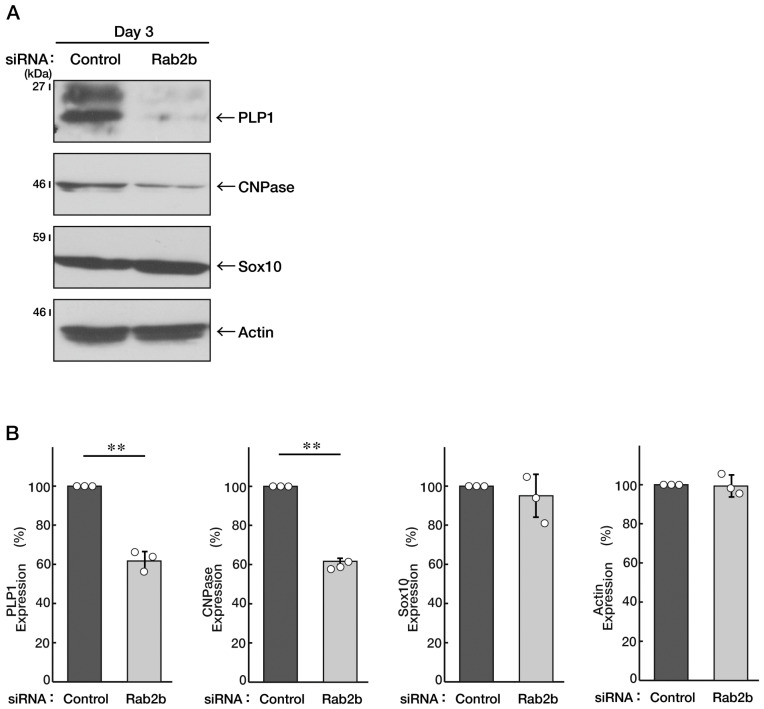
Knockdown of Rab2b decreases expression levels of marker proteins in FBD-102b cells. (**A**,**B**) FBD-102b cells were transfected with a control or Rab2b siRNAs. Transfected cells were allowed to be cultured for 3 days following the induction of differentiation. Each cell lysate was collected for an immunoblotting for an antibody against PLP1, CNPase, Sox10, or actin. Immunoreactive bands were quantitatively calculated compared to the respective controls (100%) and statistically depicted as graphs (** *p* < 0.01; n = 3 blots). White circles show percentages in the respective independent experiments.

Since white matter disorganization is observed in ASD patients [5,6,7,8], we explored whether the knockdown of Rab2b affects oligodendroglial morphological differentiation. We knocked down Rab2b in FBD-102b cells and investigated whether Rab2b is involved in the regulation of oligodendroglial morphological differentiation. Knockdown resulted in a decrease of their morphological differentiation (Figure 7A,B), consistent with the results of the decreased expression levels of marker proteins, proteolipid protein 1 (PLP1) and 2′, and 3′-cyclic nucleotide 3′-phosphodiesterase (CNPase) (Figure 8A,B). In contrast, oligodendrocyte lineage marker Sox10 and control actin proteins were comparable in control- and Rab2b-knocked-down cells.

**Figure 9:**
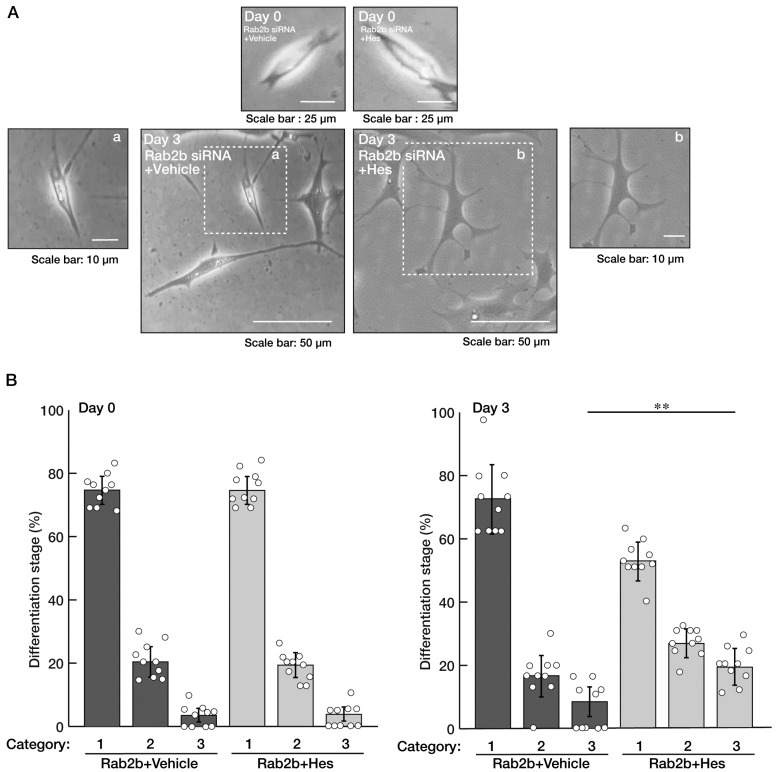
Hesperetin recovers decreased differentiated phenotypes induced by Rab2b knockdown in FBD-102b cells. (**A**,**B**) FBD-102b cells were transfected with Rab2b siRNAs and treated with a control vehicle or hesperetin (Hes, 1 μM) for 0 or 3 days. Their cell morphologies were divided into 3 categories and statistically depicted as graphs (** *p* < 0.01; n = 10 fields). White circles show percentages in the respective independent experiments.

**Figure 10:**
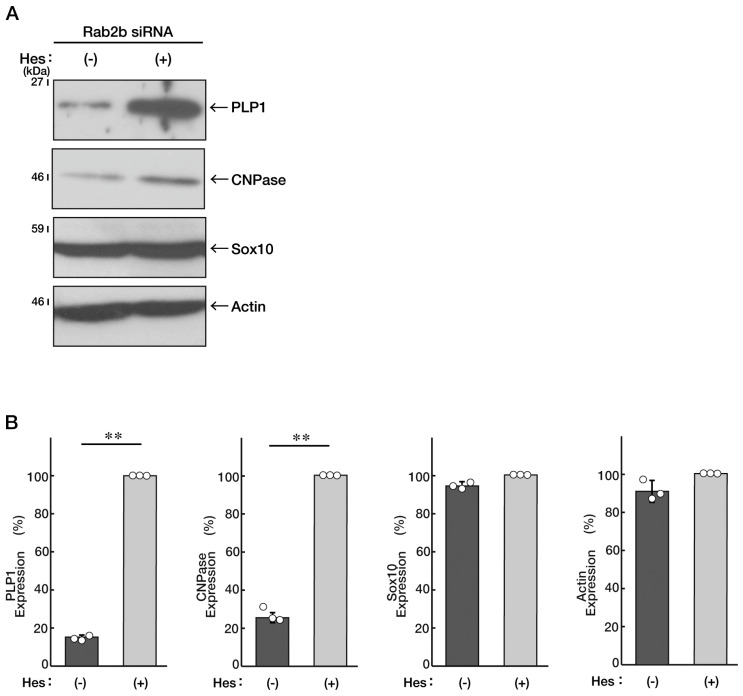
Hesperetin recovers decreased marker protein levels induced by Rab2b knockdown in FBD-102b cells. (**A**,**B**) FBD-102b cells were transfected with Rab2b siRNAs and treated with a control vehicle (minus Hes) or hesperetin (plus Hes, 1 μM) for 3 days. Each cell lysate was collected for an immunoblotting for an antibody against PLP1, CNPase, Sox10, or actin. Immunoreactive bands were quantitatively calculated compared to the respective controls (100%) and statistically depicted as graphs (** *p* < 0.01; n = 3 blots). White circles show percentages in the respective independent experiments.

We sought to determine whether hesperetin recovers the inhibitory morphological differentiation by Rab2b knockdown, similar to that in N1E-115 cells. Treatment with hesperetin in FBD-102b cells recovered the morphological differentiation, and marker protein expression levels decreased by Rab2b knockdown (Figure 9A,B and Figure 10A,B). Together with the results from N1E-115 cells, it is evident that the knockdown of Rab2b inhibits neuronal and oligodendroglial differentiation and can be recovered with treatment with hesperetin.

It is of note that, in contrast to the effects of Rab2a or Rab4b in N1E-115 cells, knockdown of Rab2a and Rab4b uniquely inhibited oligodendroglial morphological differentiation (Figures S5–S8).

### 3.4. Knockdown of Rab2b Decreases Phosphorylation Levels of Mitogen-Activated Protein Kinase, as Recovered by Hesperetin

**Figure 11:**
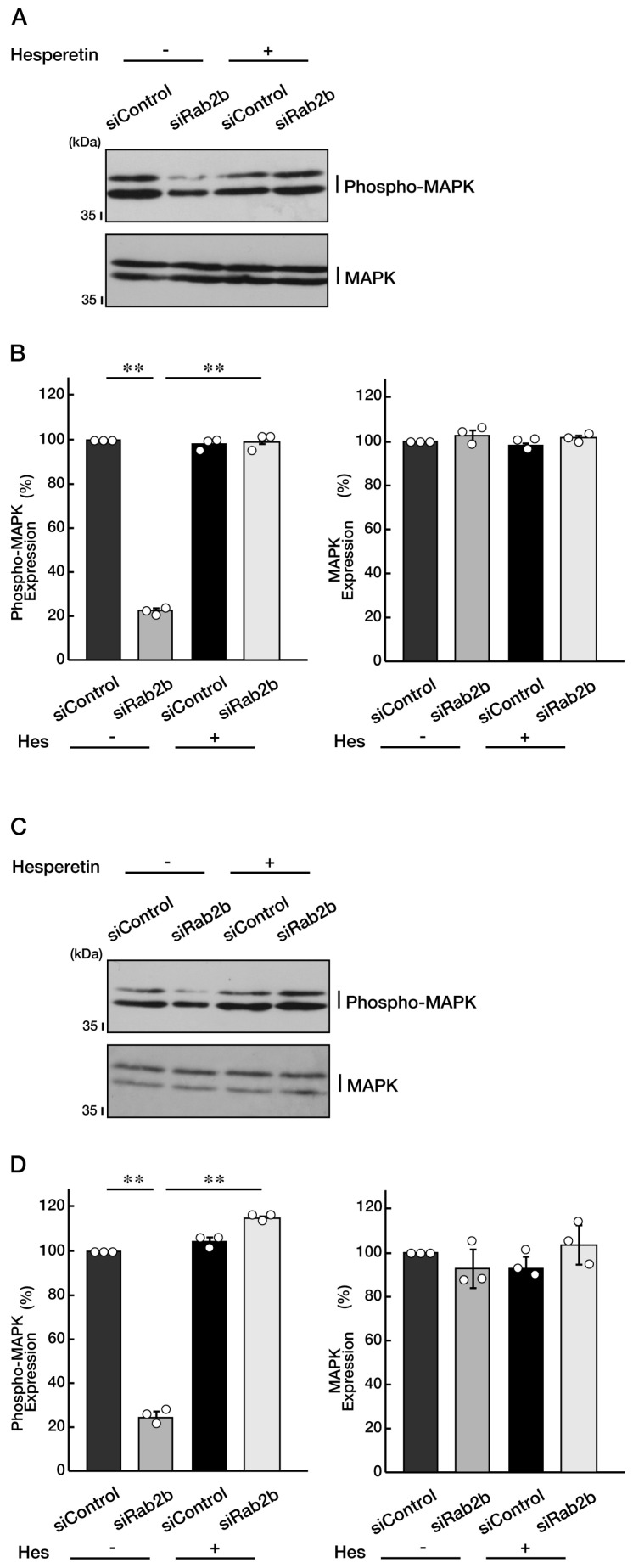
Hesperetin recovers decreased phosphorylation levels of MAPK. (**A**,**B**) N1E-115 cells were transfected with a control or Rab2b siRNAs and treated with control vehicle (minus Hes) or hesperetin (plus Hes, 1 μM) for 3 days. Each cell lysate was collected for an immunoblotting for an antibody against phospho-MAPK or MAPK. Immunoreactive bands were quantitatively calculated compared to the respective controls (100%) and statistically depicted as graphs (** *p* < 0.01; n = 3 blots). (**C**,**D**) FBD-102b cells were transfected with a control or Rab2b siRNAs and treated with a control vehicle (−) or hesperetin (1 μM) for 3 days. Each cell lysate was collected for an immunoblotting for an antibody against phospho-MAPK or MAPK. Immunoreactive bands were quantitatively calculated compared to the respective controls (100%) and statistically depicted as graphs (** *p* < 0.01; n = 3 blots). White circles show percentages in the respective independent experiments.

Mitogen-activated protein kinase (MAPK, also called extracellular signal-regulated protein kinase [ERK]) is essential for the differentiation of various cell types in nervous tissues [1,2,3,4,5,6,7,8], and its phosphorylation is important for kinase activation. Therefore, we knocked down Rab2b in N1E-115 cells and examined the levels of its phosphorylation and the protective effect of hesperetin. As a result, knockdown of Rab2b decreased the phosphorylation levels of MAPK, and the effects were recovered by the hesperetin treatment (Figure 11A,B). Similar results were obtained in the case of FBD-102b cells (Figure 11C,D), suggesting that MAPK, as the key kinase controlling cellular differentiation, is a potential target of hesperetin.

## 4. Discussion

Rab proteins are divided into members of a large family of GTP-binding proteins and control intracellular membrane trafficking [1,2,3,4]. Rab proteins undergo cycles between GTP-bound and GDP-bound states. These continuous cycles of guanine-nucleotide-binding states in Rab proteins help promote effective membrane transporting cycles, namely, intracellular vesicle budding and fusion. Therefore, it is thought that, in Rab proteins, disease-associated gene mutations causing GDP-bound states, as well as GTP-bound states, can exhibit loss-of-function, as observed in the case of peripheral nervous system neuropathies such as Charcot–Marie–Tooth disease [12,17,18]. This is in contrast to the properties of Ras GTPase family members of the prototypic signal-regulated small GTPases since their GTP-bound forms in cycles of guanine-nucleotide-binding states are only in the switch-on states [19,20]. Thus, genetic alternations of the genes encoding Rab rather than Ras proteins may have a greater variety of effects than currently expected, underscoring the relationship between genetic alternations of rab genes and defective effects on cells in various tissues and organs, including those in the nervous system [1,2,3,4,5,6,7,8].

Rab2 proteins, which are composed of Rab2b and Rab2a, are involved in the regulation of retrograde transport between the ER and the Golgi body [21,22,23]. They appear likely to regulate vesicle recycling around the Golgi body and autophagy in neuronal cells [21,22,23]. It is unclear how Rab2b is associated with ASD to bring about insufficient or defective cell morphogenesis, as observed in the organization of the neuronal and white matter tissues in ASD patients. In the present study, we show that knockdown of Rab2b, which mimics its loss-of-function effect, inhibits neuronal and oligodendroglial cell morphological changes following the induction of differentiation. In *Drosophila*, Rab2 mutant proteins are accumulated at the trans-Golgi in neuronal cell bodies, resulting in abnormal neuronal tissue morphogenesis [23]. There are also decreased numbers of synaptic terminals and, in turn, impaired neurotransmission in mutant *Drosophila* lines. It is likely that synaptic biogenesis is also linked to the proper organization of the Golgi body, depending on Rab2-controlled protein trafficking and sorting [23]; however, it remains unclear whether abnormal morphologies of *Drosophila* oligodendroglial cell-like cells are observed in the mutant line. Additionally, in adult *Drosophila*, Rab2 proteins positively regulate the transport of trans-Golgi-derived neuropeptide-containing dense core vesicles, endosomes, and lysosomes in neuronal axons [4]. Rab2b is essential for maintaining the Golgi morphology since knockdown of its specific effector, Golgi-associated Rab2b interactor-like 4 (GARIN4, also called FAM71A), results in the fragmentation of the Golgi body in human cells other than neuronal cells [22]. Despite the unknown role of Rab2b in cells of mature neuronal tissues, including oligodendroglial cells, it is conceivable that functional deficiency of Rab2b proteins can lead to abnormal morphologies of the Golgi body and inhibition of the related intracellular transporting system, resulting in neuronal tissue and white matter disorganization.

While Rab2a functionally resembles Rab2b, it has been demonstrated that Rab2a is specifically required for autolysosome formation and, in turn, autophagosome clearance in *Drosophila* Schneider 2 (S2) cells and some mammalian cells [22]. It is of note that the knockdown of Rab2a has an effect only on oligodendroglial cells, not on neuronal cells. Similar to GARIN4 as the Rab2b-specific effector [22], there may be an as-yet-unknown Rab2a-specific effector protein. The putative proteins may be specifically involved in autolysosome formation since the vesicle transport system around the lysosome plays a key role in morphological differentiation in oligodendroglial cells [24,25]. Actually, a loss-of-function mutation of vacuolar protein sorting 11 (VPS11), as the core subunit of complexes of class C core vacuole/endosome tethering (CORVET) and homotypic fusion and vacuole protein sorting (HOPS), specifically causes hypomyelinating leukodystrophy 11 (HLD11), which is a genetic oligodendroglial cell disease [26]. It may be important to clarify the specific organelle role of Rab2a in oligodendroglial cells, judging by the relationship between Rab2a and morphological differentiation in oligodendroglial cells.

As with Rab2a, Rab4b specifically contributes to oligodendroglial cell morphological differentiation. Although Rab4b has been shown to govern endocytic recycling [27,28,29,30], it is known that Rab4b is perfectly colocalized with the mammalian MON2 homolog known as the regulator of endosome-to-Golgi trafficking [27,28,29,30]. It is likely that Rab4b couples the recycling of endocytic vesicles to transporting around the Golgi body. However, if Rab4b, acting like Rab2b, could mediate a morphological change of the Golgi body, its knockdown might affect morphological differentiation in certain neuronal cells as well. The precise relationship between the proper organization of the Golgi body and cell morphogenesis in neuronal cells remains to be determined. In the future, further studies of Rab4b in the cell biological research fields will allow us to establish the common intracellular role of Rab4b.

It is known that Rab2b is related to the growth of cervical cancer cells [31]. Expression levels of methyltransferase-like 3 (METTL3) are increased in cervical tumor tissues. METTL3 participates in the mRNA stability of Rab2, promoting the growth of cervical cancer cells [31]. In addition, in pancreatic cancer cells, microRNA (miR)-448 is downregulated. MiR-448 itself promoted cell cycle arrest and apoptosis in pancreatic cancer cells [32]. Since its target is Rab2, Rab2 is thought to regulate pancreatic cancer cell growth [32]. Although it remains unclear whether Rab2 is related to promoting brain tumors, further research will clarify the precise role of Rab2b in tumor progression.

Hesperidin is soluble in water due to the sugar moiety in its structure and upon digestion, releases the aglycon hesperetin. Hesperetin is an essential functional group of a citrus flavonoid that has multiple cellular protective effects, including on brain tissues, and activates various kinases, including MAPK as well as Akt kinase [13,14,15,16]. Knockdown of Rab2b decreases both neuronal and oligodendroglial cell morphological differentiation with decreased MAPK phosphorylation. In both types of cells, the possible underlying mechanism contains MAPK since MAPK is indispensable for neuronal and glial cell development, and its failure is strictly associated with various diseases, including neuronal disorders [33]. Since treatment with hesperetin can increase MAPK phosphorylation to stimulate its activity in both types of cells, it is possible that hesperetin recovers decreased MAPK phosphorylation to promote cell morphological differentiation.

Herein we demonstrate that the knockdown of ASD-associated Rab2b inhibits neuronal and oligodendroglial cell morphological differentiation with decreased marker proteins. Their defective morphological differentiation may, at least in part, reflect the neuronal and white matter disorganization observed in ASD patients. We have also described that hesperetin, a citrus flavonoid, recovers these defective changes (see Figure S9, schematic diagram). Further studies will lead to an improved understanding of both the detailed mechanisms by which Rab2b mediates cell morphological differentiation and also the molecular mechanisms underlying the protective effects of hesperetin. It is of note that hesperetin has effects on neuronal and oligodendroglial cells. Hesperetin and probable hesperetin derivative(s) might be applied in clinical trials for ASD. Chemical drugs, including psychostimulants, antipsychotic drugs, antidepressants, α2-adrenergic receptor agonists, cholinesterase inhibitors, NMDA receptor antagonists, and mood stabilizers are available for pharmacological therapies for ASD [34]; however, it is still unknown whether they have effects on both neuronal and oligodendroglial cells. In addition, future research may clarify why Rab2a and Rab4b specifically play key roles in oligodendroglial cell morphological differentiation. Studies along these lines may lead to the development of chemical compounds or biopharmaceuticals for ASD therapeutic target molecules around signaling through Rab2b and Rab2 subfamily molecules, as might be achieved with hesperetin and hesperetin derivative(s).

## Figures and Tables

**Table 1:** List of antibodies.

Reagent or Source	Company or Source	Cat. No.	Lot. No.	Concentration Used
Antibodies				
Anti-proteolipid protein 1 (PLP1)	Atlas Antibodies (Stockholm, Sweden)	HPA004128	B115828	Immunoblotting (IB), 1/500
Anti-2′, 3′-cyclic nucleotide 3′-phosphodiesterase (CNPase)	Santa Cruz Biotechnology (Santa Cruz, CA, USA)	sc-166559	F1514	IB, 1/500
Anti-SRY-related HMG-box protein 10 (SOX10)	Santa Cruz Biotechnology	sc-365692	F1621	IB, 1/100
Anti-Actin (pan-b type)	MBL (Tokyo, Japan)	M177-3	008	IB, 1/5000
Anti-growth-associated protein 43 (GAP43)	Santa Cruz Biotechnology	sc-17790	J0920	IB, 1/8500
Anti-Tubulin a chain 1a (TUBA1A, also called TUBA)	MBL	M175-3	001	IB, 1/500,000
Anti-Tau	Santa Cruz Biotechnology	sc-21796	H2721	IB, 1/250
Anti-IgG (H+L chain) (mouse) pAb-HRP	MBL	330	366	IB, 1/5000
Anti-IgG (H+L chain) (rabbit) pAb-HRP	MBL	458	353	IB, 1/5000

**Table 2:** List of chemicals.

Reagent or Source	Company or Source	Cat. No.	Lot. No.	Concentration Used
Key Chemicals				
Hesperetin	Santa Cruz Biotechnology	sc-202647	D1921	1 or 10 μM
Dimethyl sulfoxide (DMSO, control vehicle)	FUJIFILM Wako Pure Chemical Corporation (Tokyo, Japan)	047-29353	CDN0170	0.10%

**Table 3:** List of reagents.

Reagent or Source	Company or Source	Cat. No.	Lot. No.	Concentration Used
Key Reagents				
ScreenFect TM siRNA Transfection Reagent	FUJIFILM Wako Pure Chemical Corporation	292-75013	CAM0357	According to manufacturer’s instructions
ScreenFect TM Dilution Buffer	FUJIFILM Wako Pure Chemical Corporation	194-18181	SKF5794	According to manufacturer’s instructions
ImmunoStar Zeta	FUJIFILM Wako Pure Chemical Corporation	295-72404	LEN3912	According to manufacturer’s instructions
Chemi-Lumi One Ultra	Nacalai Tesque (Kyoto, Japan)	11644-24	L1P7669	According to manufacturer’s instructions
Skim Milk Powder	FUJIFILM Wako Pure Chemical Corporation	190-12865	SKG4901	According to manufacturer’s instructions
Western Blotting (WB) Stripping Solution	Nacalai Tesque	05364-55	L5M5218	According to manufacturer’s instructions
Gflex DNA Polymerase	TaKaRa Bio (Shiga, Japan)	R060A	AL80564A	According to manufacturer’s instructions
2×Gflex PCR Buffer (Mg^2+^, dNTP plus)	TaKaRa Bio	R060A	AL80564A	According to manufacturer’s instructions
ISOGEN	Nippon Gene (Tokyo, Japan)	311-02501	75009K	According to manufacturer’s instructions
Sample Buffer Solution (plus 2-Mercaptoethanol) (4-fold mixtures)	FUJIFILM Wako Pure Chemical Corporation	191-13272	WDP4995	According to manufacturer’s instructions
Pre-stained Protein Markers (Broad Range) for SDS-PAGE	Nacalai Tesque	02525-35	L9M9989	According to manufacturer’s instructions
5×Prime Script Master Mix	TaKaRa Bio	RR036A	AIE0440A	According to manufacturer’s instructions

**Table 4:** List of cell lines.

Reagent or Source	Company or Source	Cat. No.	Lot. No.	Concentration Used
Cell Lines				
FBD-102b cells (mouse cells)	Dr. Yasuhiro Tomo-oka (Riken, Saitama, Japan and Tokyo University of Science, Chiba, Japan)	N/A	N/A	N/A
N1E-115 cells (mouse cells)	Dr. Daisuke Shiokawa (Tokyo Science University, Chiba, Japan)	N/A	N/A	N/A

**Table 5:** List of DNA primers.

Reagent or Source	Company or Source	Cat. No.	Lot. No.	Concentration Used
DNA Primers (5′ to 3′)				
Sense primer for actin (internal control) ATGGATGACGATATCGCTGCGCTGGTC Antisense primer for actin (internal control) CTAGAAGCACTTGCGGTGCACGATGGAG	This manuscript	N/A	N/A	0.5 μM
Sense primer for Rab2a ATGGCGTACGCCTATCTCTTCAAGT Antisense primer for Rab2a TCAACAGCAGCCTCCCCCT	This manuscript	N/A	N/A	0.5 μM
Sense primer for Rab2b ATGACTTACGCTTATCTCTTCAAGTACATCATCATC Antisense primer for Rab2b TCAGCAGCAGCCAGAGTCAG	This manuscript	N/A	N/A	0.5 μM
Sense primer for Rab4a CCGGGATCCATGGCGCAGACCGCCATG Antisense primer for Rab4a CCGGGATCCCTAGCAGCCACACTCCTGTGCAC	This manuscript	N/A	N/A	0.5 μM
Sense primer for Rab4b CCGAGATCTATGGCCGAGACCTACGACTTCCTC Antisense primer for Rab4b CCGAGATCTTCAGCAGCCACAGGGCTGAG	This manuscript	N/A	N/A	0.5 μM

**Table 6:** List of siRNAs.

Reagent or Source	Company or Source	Cat. No.	Lot. No.	Concentration Used
siRNA Sequences (5′ to 3′)				
Sense chain for siLuciferase (Control siRNA) GCCAUUCUAUCCUCUAGAG-dTdT Antisense chain for siLuciferase (Control siRNA) CUCUAGAGGAUAGAAUGGC-dTdT	This manuscript	N/A	N/A	N/A
Sense chain for siRab2a-87th GAGGUUUCAGCCGGUGCAU-dTdT Antisense chain for siRab2a-87th AUGCACCGGCUGAAACCUC-dTdT	This manuscript	N/A	N/A	N/A
Sense chain for siRab2a-269th GGAGAGACACGUUCAACCA-dTdT Antisense chain for siRab2a-269th UGGUUGAACGUGUCUCUCC-dTdT	This manuscript	N/A	N/A	N/A
Sense chain for siRab2b-87th GCGGUUCCAGCCCGUGCAC-dTdT Antisense chain for siRab2b-87th GUGCACGGGCUGGAACCGC-dTdT	This manuscript	N/A	N/A	N/A
Sense chain for siRab2b-196th GAGUCCUUCCGUUCUAUCA-dTdT Antisense chain for siRab2b-196th UGAUAGAACGGAAGGACUC-dTdT	This manuscript	N/A	N/A	N/A
Sense chain for siRab4b-96th GUUCAAACAGGACUCCAAC-dTdT Antisense chain for siRab4b-96th GUUGGAGUCCUGUUUGAAC-dTdT	This manuscript	N/A	N/A	N/A
Sense chain for siRab4b-165th GACUGUGAAACUACAGAUU-dTdT Antisense chain for siRab4b-165th AAUCUGUAGUUUCACAGUC-dTdT	This manuscript	N/A	N/A	N/A
Sense chain for siRab4b-366th GAAGGACCUGGAUCCCGAG-dTdT Antisense chain for siRab4b-366th CUCGGGAUCCAGGUCCUUC-dTdT	This manuscript	N/A	N/A	N/A

## Data Availability

Not applicable.

## Supplementary Materials

The following supporting information can be downloaded at: https://www.mdpi.com/article/10.3390/neurolint15010025/s1, Figure S1: RT-PCRs for Rab2a and Rab2b in N1E-115 cells; Figure S2: RT-PCRs for Rab4b in N1E-115 cells; Figure S3: Knockdown of Rab4b does not significantly change neuronal morphological differentiated phenotypes; Figure S4: Knockdown of Rab4b does not significantly change expression levels of neuronal differentiation marker proteins; Figure S5: Knockdown of Rab2a decreases morphologically differentiated phenotypes in FBD-102b cells; Figure S6: Knockdown of Rab2a decreases expression levels of marker proteins in FBD-102b cells; Figure S7: Knockdown of Rab4b decreases morphologically differentiated phenotypes in FBD-102b cells; Figure S8: Knockdown of Rab4b decreases expression levels of marker proteins in FBD-102b cells; Figure S9: Schematic diagram for the potential roles of Rab2b and hesperetin in neuronal and oligodendroglial differentiation; Figure S10: Full-size gels for immunoblots in the Figure 2, Figure 4 and Figure 6; Figure S11: Full-size gels for immunoblots in the Figure 8, Figure 10 and Figure 11; Figure S12: Full-size gels for immunoblots in supplemental figures.
