# Supplementary material for: Hesperetin Ameliorates Inhibition of Neuronal and Oligodendroglial Cell Differentiation Phenotypes Induced by Knockdown of Rab2b, an Autism Spectrum Disorder-Associated Gene Product
Source: Neurol Int. 2023 Mar 10;15(1):371–91. doi: 10.3390/neurolint15010025 (PMC10057161; doi:10.3390/neurolint15010025)
Supplement: Supplementary file 1 [file neurolint-15-00025-s001.zip › 23 03 supplemental files/01 Supplemental legends.pdf]

Supplemental figure legends

Figure S1. RT-PCRs for Rab2a and Rab2b in N1E-115 cells. Total RNAs from N1E-115 cells were isolated and used for Rab2a/b RT-PCRs before agarose gel electrophoresis. Amplification of actin is shown as the control. Sequences of Rab2/b siRNAs are also shown. For experiments, siRNAs of 269th and 196th target sequences for Rab2a and Rab2b, respectively, were used.

Figure S2. RT-PCRs for Rab4b in N1E-115 cells. Total RNAs from N1E-115 cells were isolated and used for Rab4b RT-PCR before agarose gel electrophoresis. Amplification of actin is shown as the control. Sequences of Rab4b siRNAs are also shown. For experiments, siRNA of 96th target sequence for Rab4b was used. RT-PCR for Rab4a was performed using total RNAs from N1E-115 cells and mouse brain and an Rab4a band was under detection levels.

Figure S3. Knockdown of Rab4b does not significantly change neuronal morphological differentiated phenotypes. (A, B) N1E-115 cells were transfected with control or Rab4b siRNAs. Transfected cells were allowed to be cultured for 0 or 3 days following the induction of differentiation. Their cell morphologies were divided into 3 categories and statistically depicted as graphs (n = 10 fields).

Figure S4. Knockdown of Rab4b does not significantly change expression levels of neuronal differentiation marker proteins. (A, B) N1E-115 cells were transfected with control or Rab4b siRNAs. Transfected cells were allowed to be cultured for 3 days

following the induction of differentiation. Each cell lysate was collected for an immunoblotting for an antibody against GAP43, TUBA, Tau, or actin. Immunoreactive bands were quantitatively calculated compared to the respective controls (100%) and statistically depicted as graphs (n = 3 blots).

Figure S5. Knockdown of Rab2a decreases morphologically differentiated phenotypes in FBD-102b cells. (A, B) FBD-102b cells were transfected with control or Rab2a siRNAs. Transfected cells were allowed to be cultured for 0 or 3 days following the induction of differentiation. Their cell morphologies were divided into 3 categories and statistically depicted as graphs (** $p < 0.01$; n = 10 fields).

Figure S6. Knockdown of Rab2a decreases expression levels of marker proteins in FBD-102b cells. (A, B) FBD-102b cells were transfected with control or Rab2a siRNAs. Transfected cells were allowed to be cultured for 3 days following the induction of differentiation. Each cell lysate was collected for an immunoblotting for an antibody against PLP1, CNPase, Sox10, or actin. Immunoreactive bands were quantitatively calculated compared to the respective controls (100%) and statistically depicted as graphs (** $p < 0.01$; n = 3 blots).

Figure S7. Knockdown of Rab4b decreases morphologically differentiated phenotypes in FBD-102b cells. (A, B) FBD-102b cells were transfected with control or Rab4b siRNAs. Transfected cells were allowed to be cultured for 0 or 3 days following

the induction of differentiation. Their cell morphologies were divided into 3 categories and statistically depicted as graphs (** $p < 0.01$; $n = 10$ fields).

Figure S8. Knockdown of Rab4b decreases expression levels of marker proteins in FBD-102b cells. (A, B) FBD-102b cells were transfected with control or Rab4b siRNAs. Transfected cells were allowed to be cultured for 3 days following the induction of differentiation. Each cell lysate was collected for an immunoblotting for an antibody against PLP1, CNPase, Sox10, or actin. Immunoreactive bands were quantitatively calculated compared to the respective controls (100%) and statistically depicted as graphs (** $p < 0.01$; $n = 3$ blots).

Figure S9. Schematic diagram for the potential roles of Rab2b and hesperetin in neuronal and oligodendroglial differentiation. Neuronal and oligodendroglial cells indicate blue and red lines, respectively. Arrow also indicates induction of differentiation.

Figure S10. Full-size gels for immunoblots in the Figures 2, 4, and 6.

Figure S11. Full-size gels for immunoblots in the Figures 8, 10, and 11.

Figure S12. Full-size gels for immunoblots in supplemental figures.
