# Supplementary figures and images for: Hesperetin Ameliorates Inhibition of Neuronal and Oligodendroglial Cell Differentiation Phenotypes Induced by Knockdown of Rab2b, an Autism Spectrum Disorder-Associated Gene Product
Source: Neurol Int. 2023 Mar 10;15(1):371–91. doi: 10.3390/neurolint15010025 (PMC10057161; doi:10.3390/neurolint15010025)

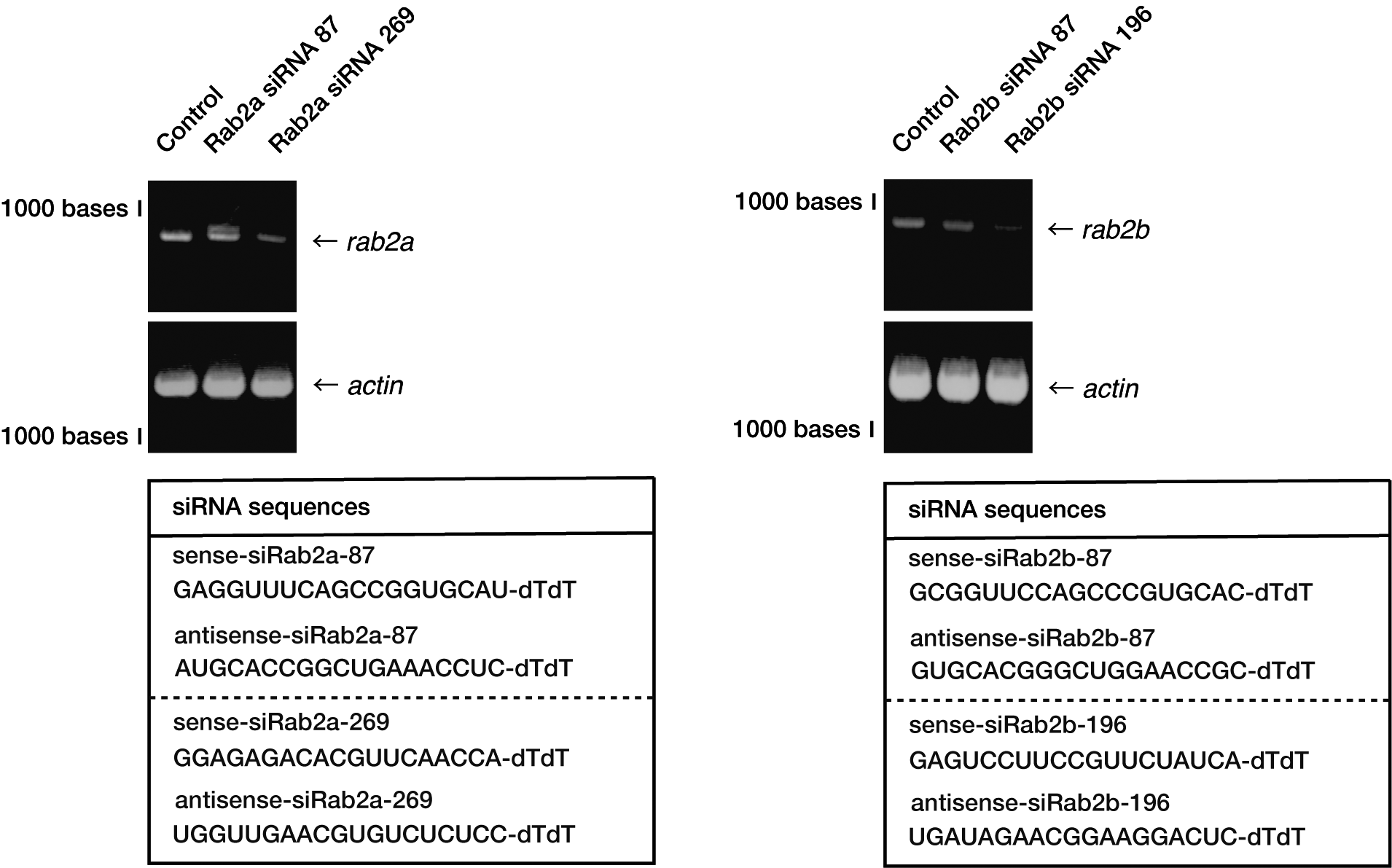

Supplement: Supplementary file 1 [file neurolint-15-00025-s001.zip › 23 03 supplemental files/Figure S1.tif]

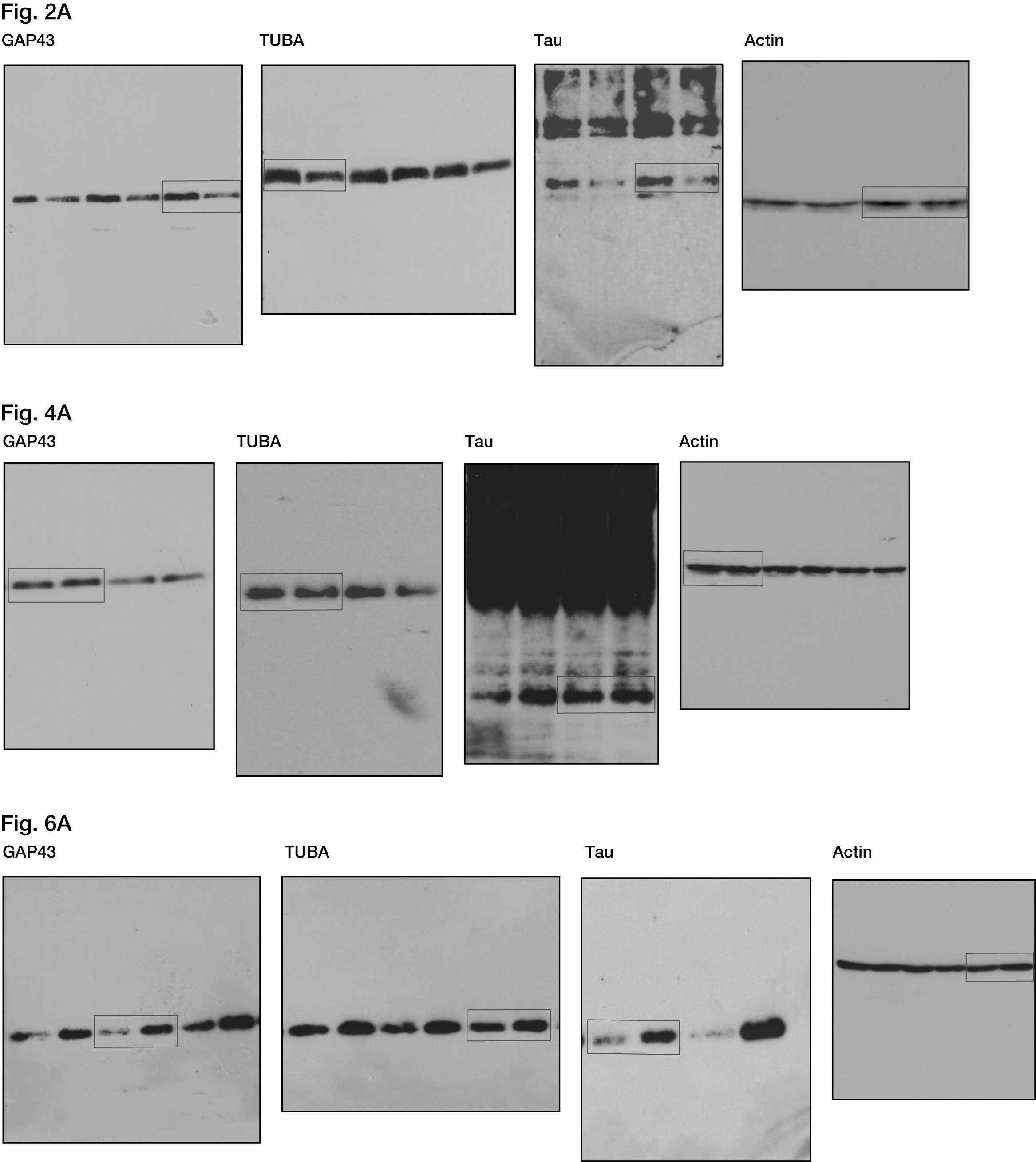

Supplement: Supplementary file 1 [file neurolint-15-00025-s001.zip › 23 03 supplemental files/Figure S10.tif]

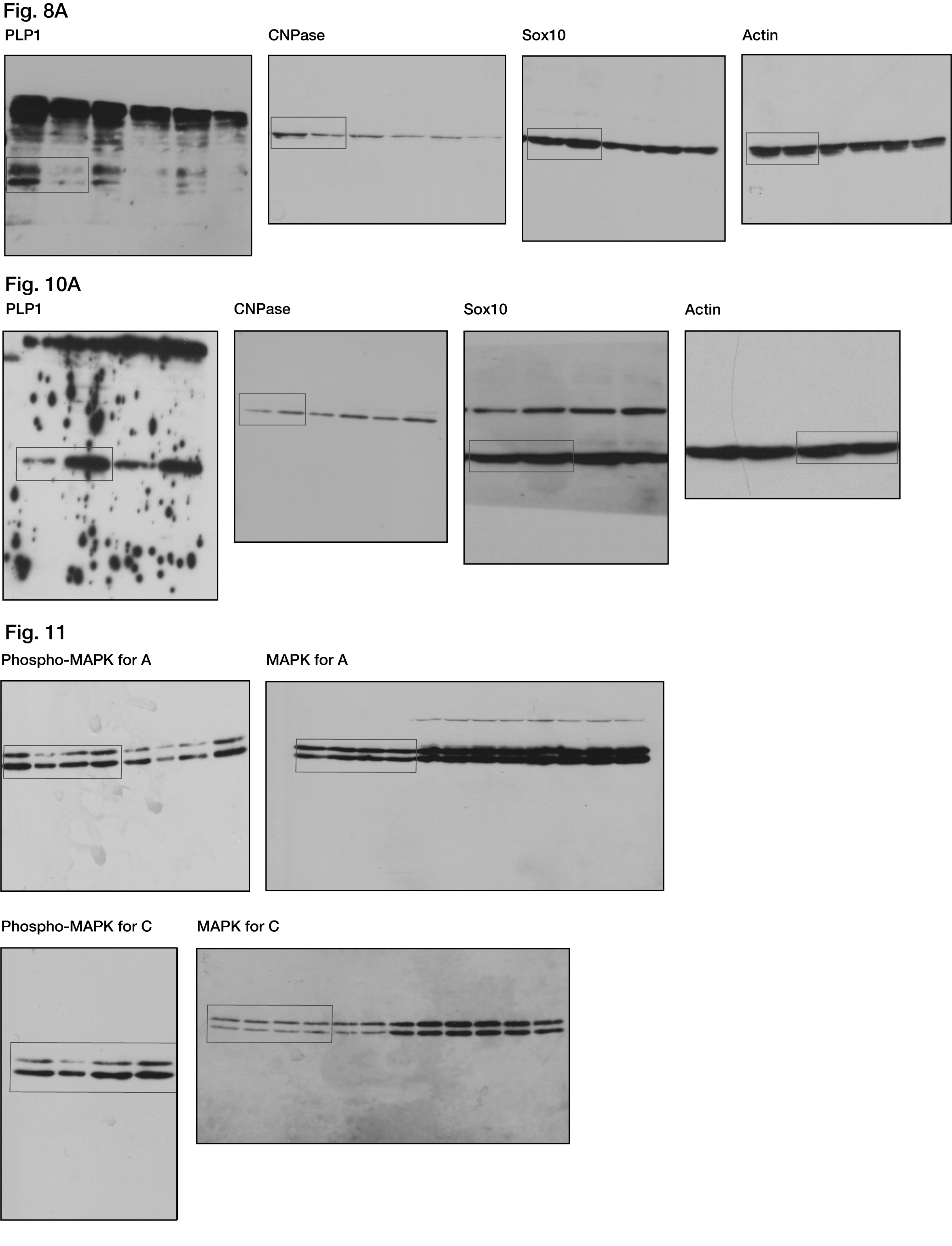

Supplement: Supplementary file 1 [file neurolint-15-00025-s001.zip › 23 03 supplemental files/Figure S11.tif]

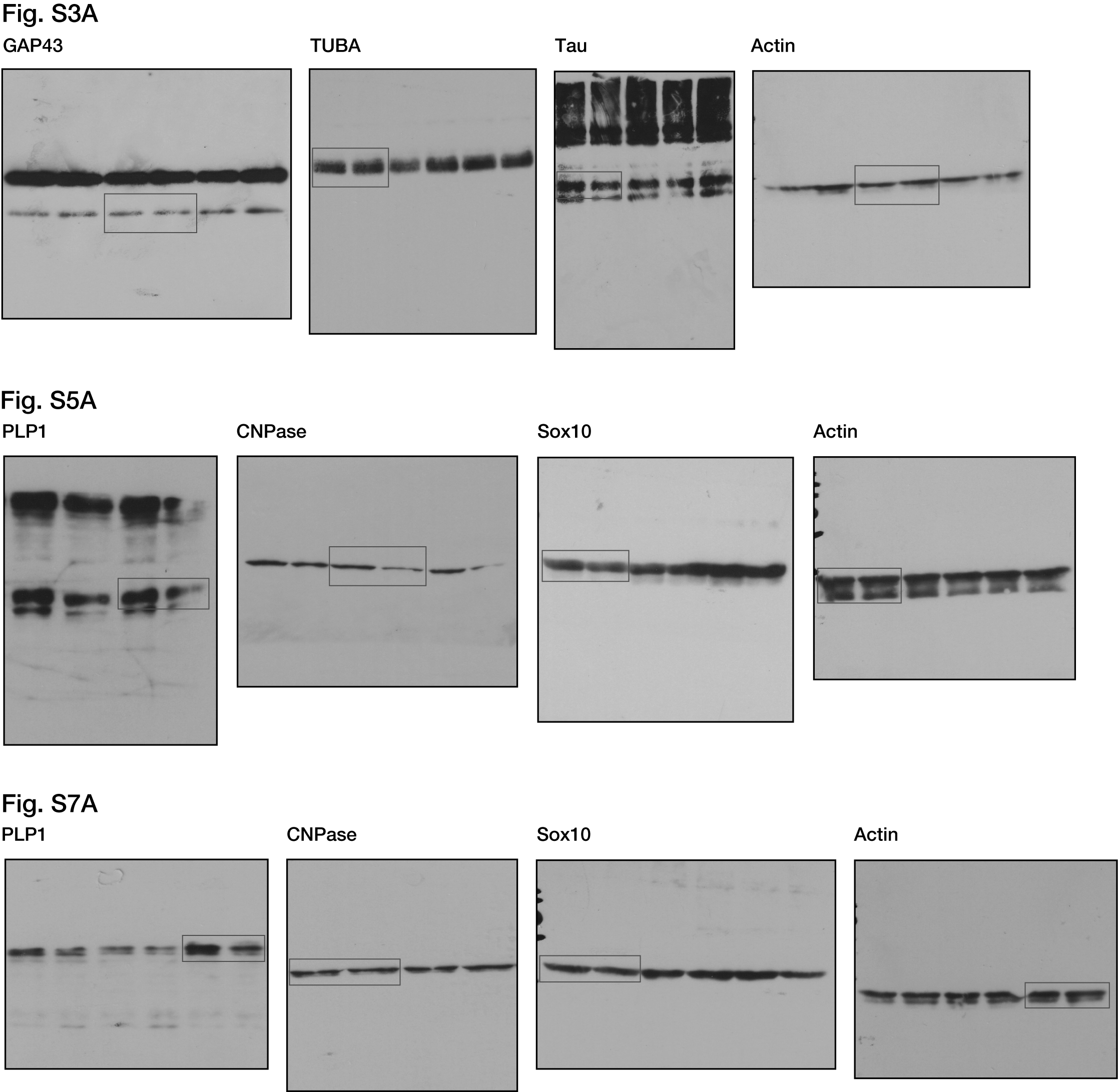

Supplement: Supplementary file 1 [file neurolint-15-00025-s001.zip › 23 03 supplemental files/Figure S12.tif]

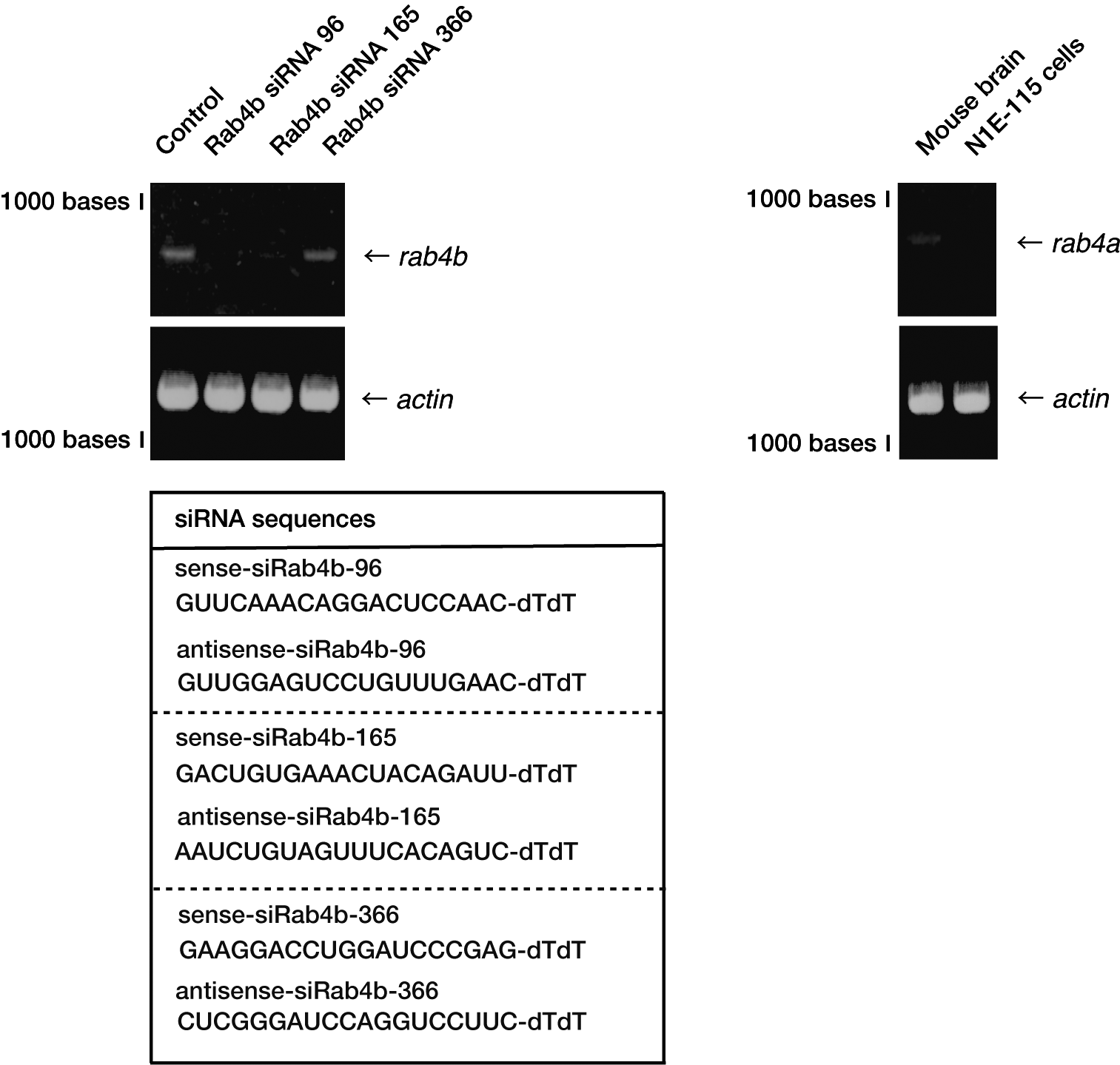

Supplement: Supplementary file 1 [file neurolint-15-00025-s001.zip › 23 03 supplemental files/Figure S2.tif]

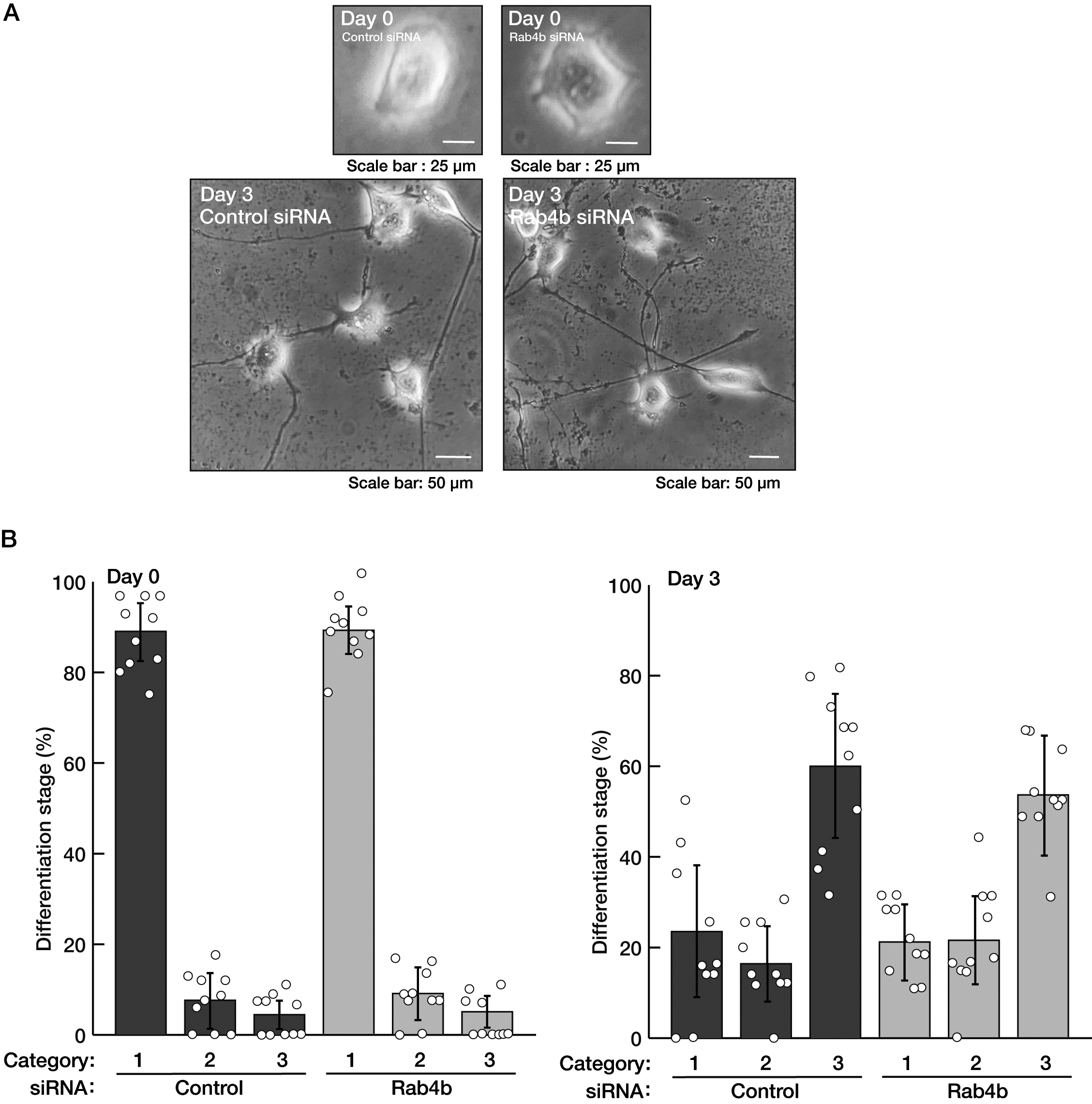

Supplement: Supplementary file 1 [file neurolint-15-00025-s001.zip › 23 03 supplemental files/Figure S3.tif]

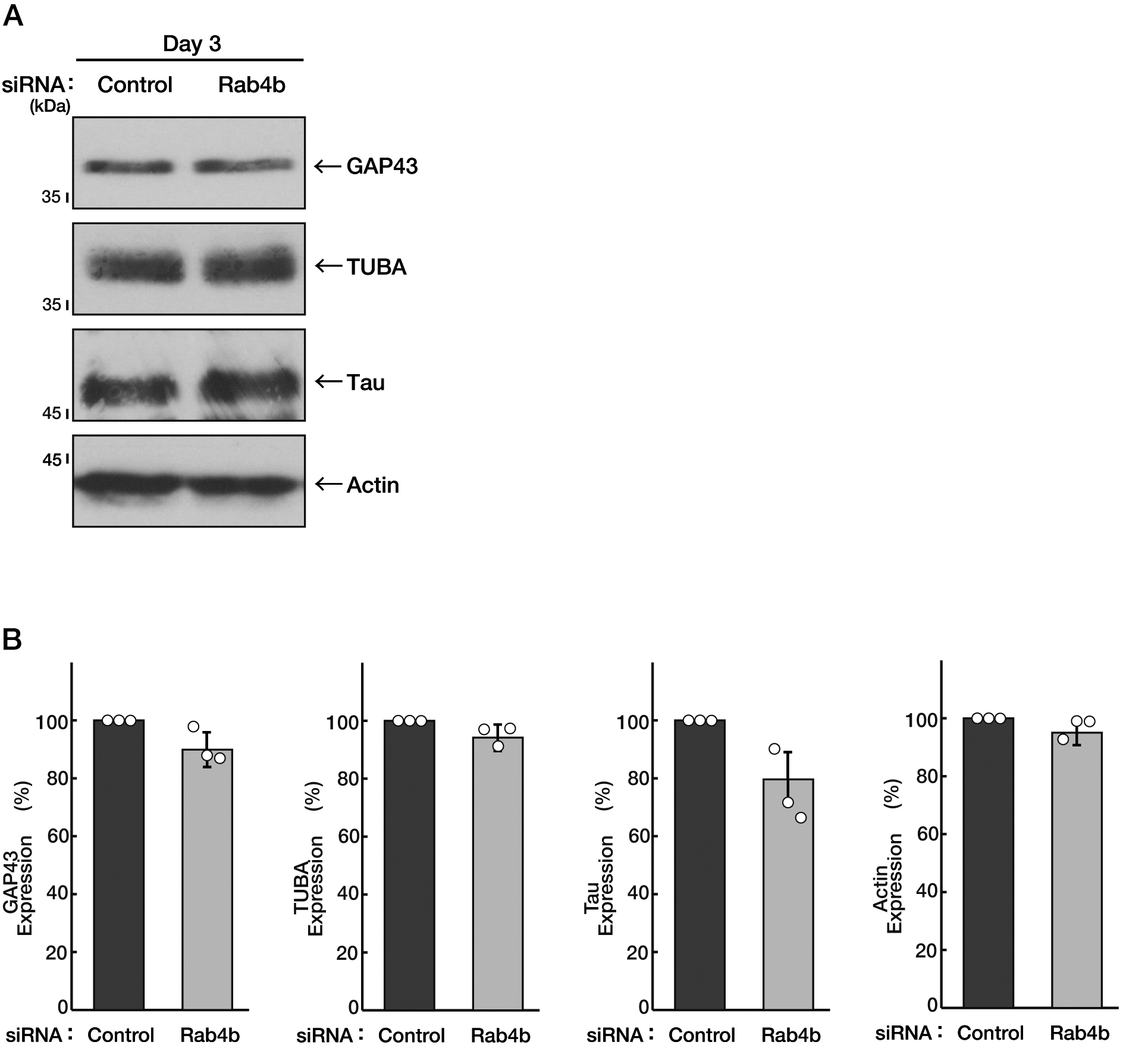

Supplement: Supplementary file 1 [file neurolint-15-00025-s001.zip › 23 03 supplemental files/Figure S4.tif]

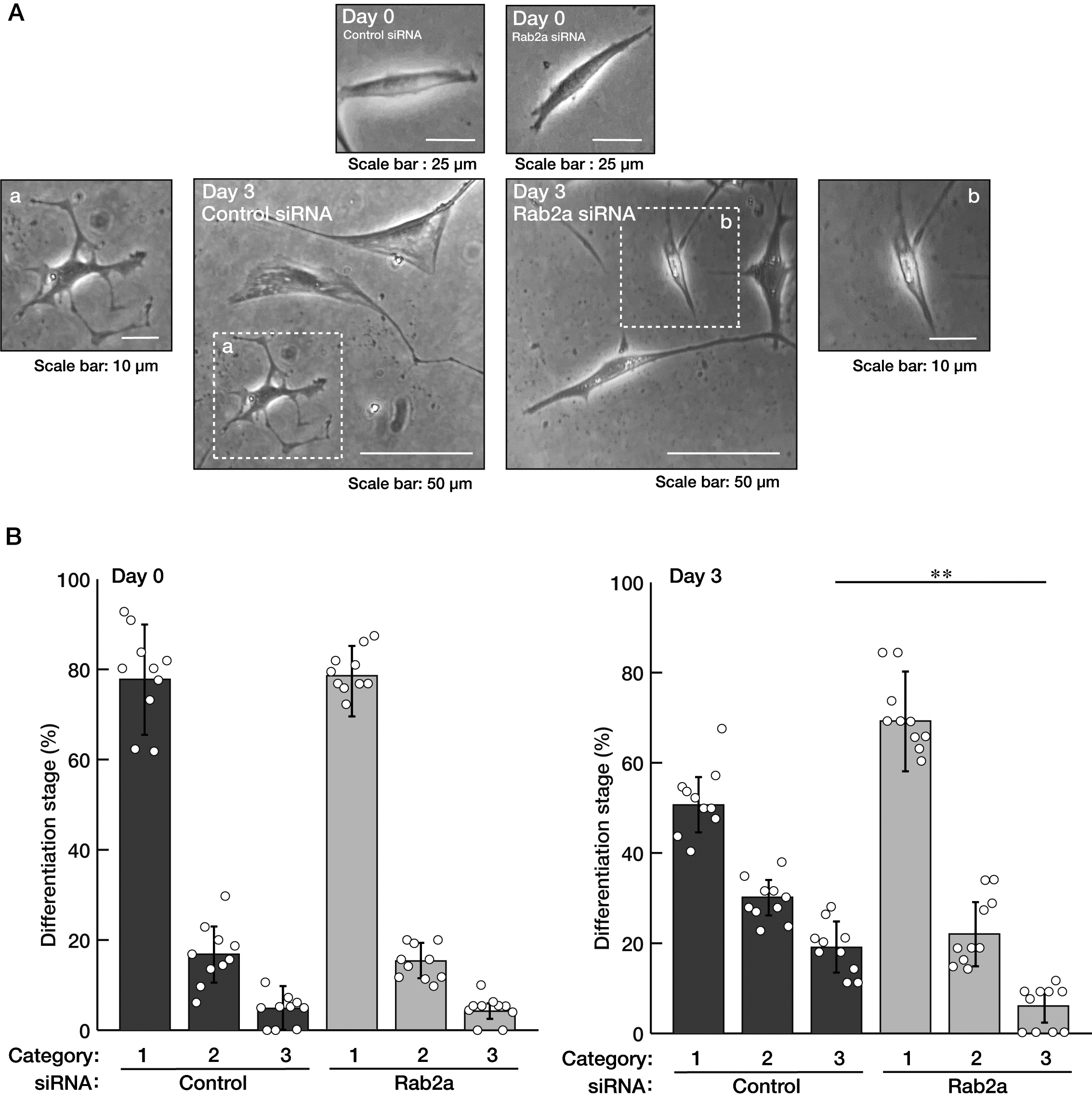

Supplement: Supplementary file 1 [file neurolint-15-00025-s001.zip › 23 03 supplemental files/Figure S5.tif]

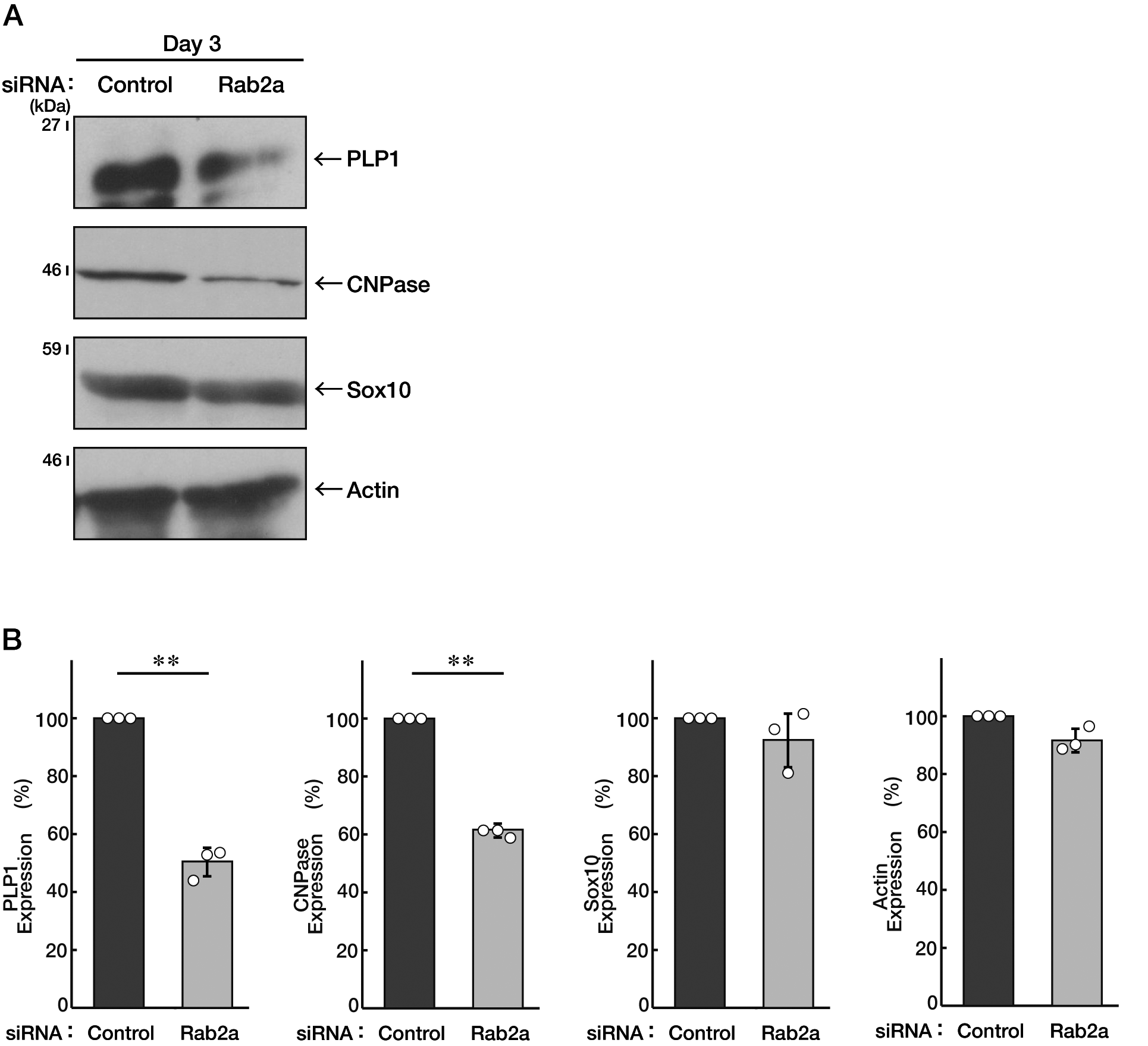

Supplement: Supplementary file 1 [file neurolint-15-00025-s001.zip › 23 03 supplemental files/Figure S6.tif]

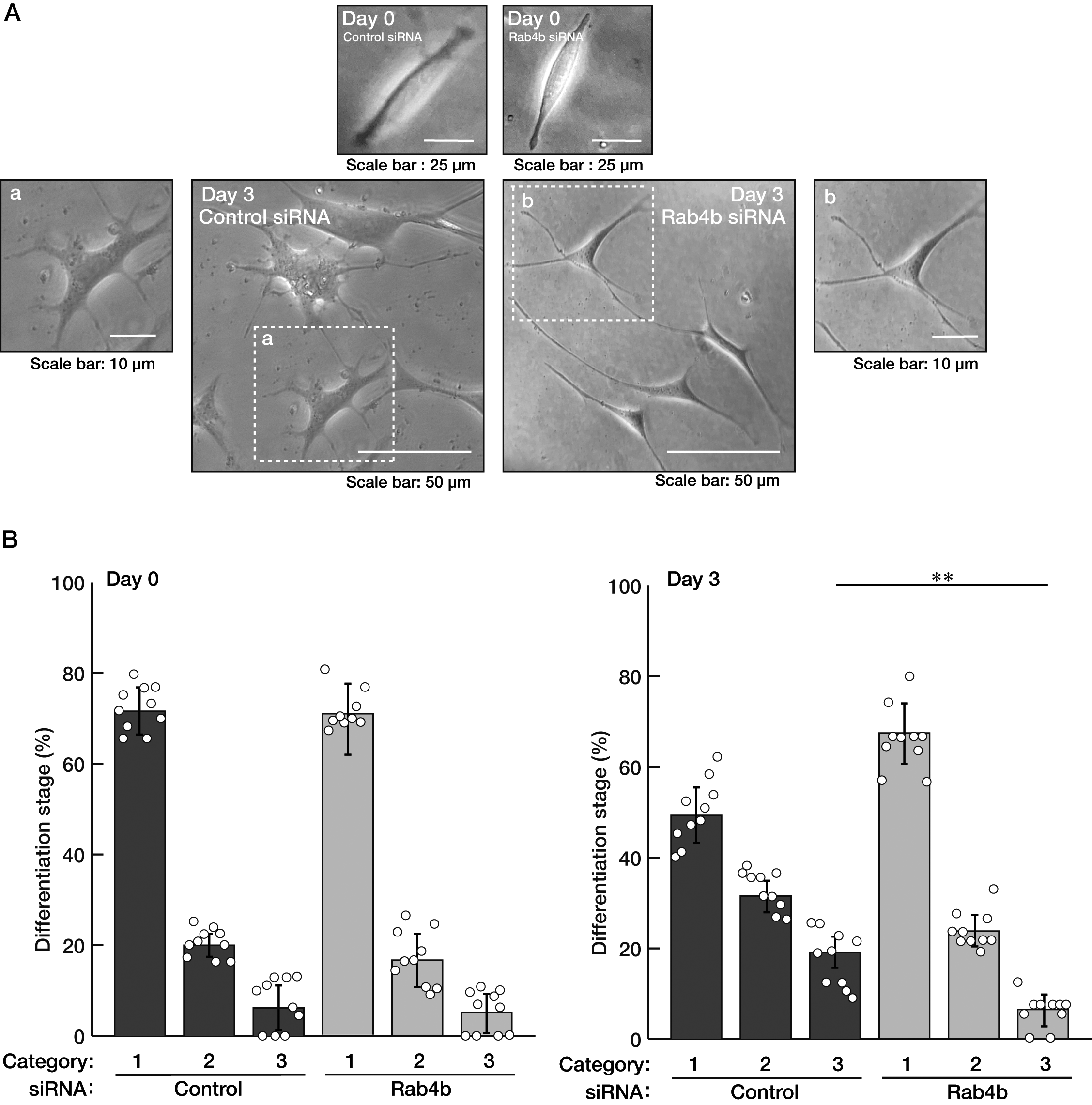

Supplement: Supplementary file 1 [file neurolint-15-00025-s001.zip › 23 03 supplemental files/Figure S7.tif]

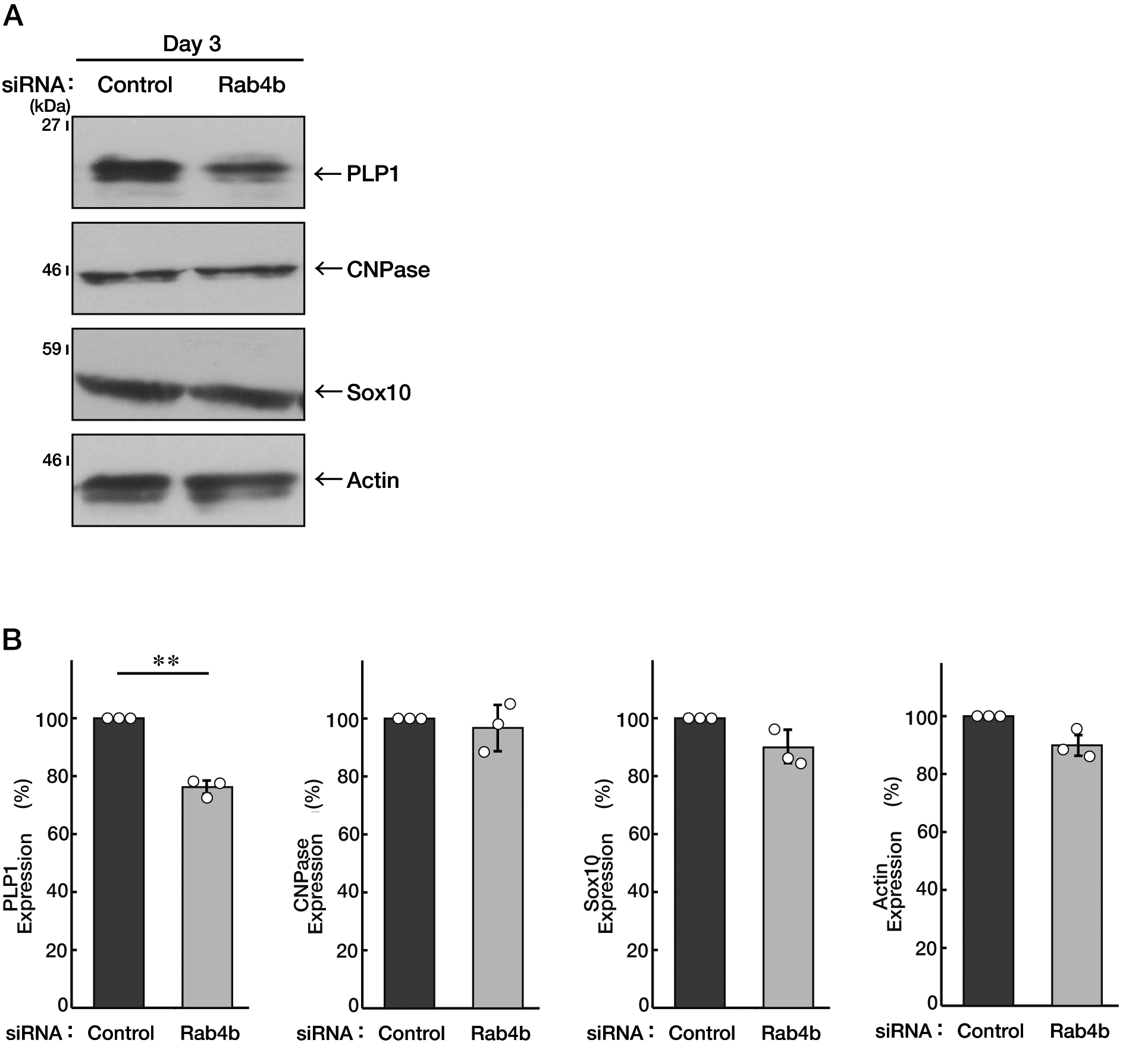

Supplement: Supplementary file 1 [file neurolint-15-00025-s001.zip › 23 03 supplemental files/Figure S8.tif]

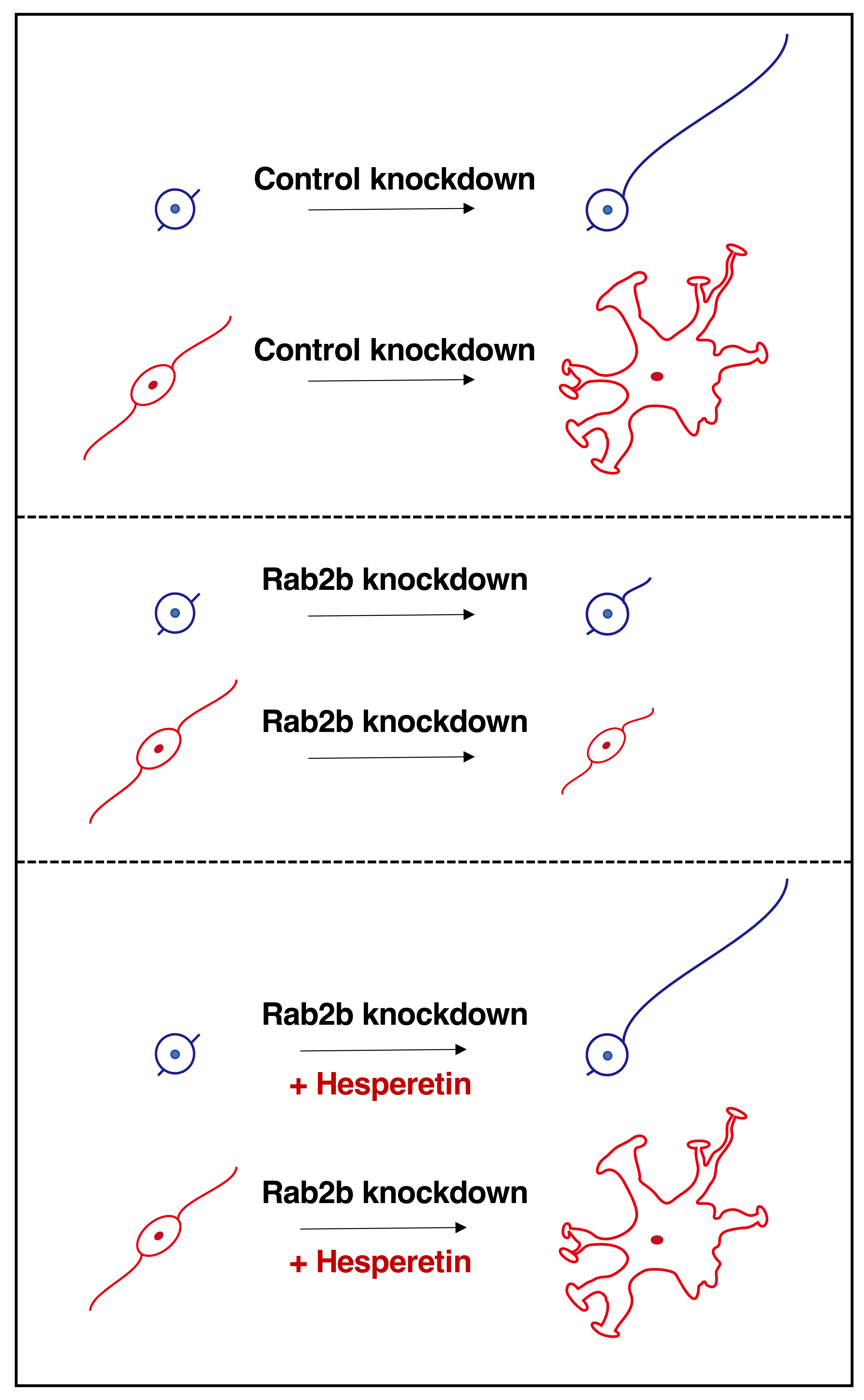

Supplement: Supplementary file 1 [file neurolint-15-00025-s001.zip › 23 03 supplemental files/Figure S9.tif]
